# Bio‐Inspired Strategy for Radiation‐Based Thermal Management and Utilization

**DOI:** 10.1002/advs.202502851

**Published:** 2025-06-24

**Authors:** Hyung Rae Kim, Won Bae Han, Se‐Yeon Heo, Yun Su Go, Suk‐Won Hwang, Young Min Song

**Affiliations:** ^1^ School of Electrical Engineering and Computer Science Gwangju Institute of Science and Technology (GIST) Gwangju 61005 Republic of Korea; ^2^ KU‐KIST Graduate School of Converging Science and Technology Korea University Seoul 02841 Republic of Korea; ^3^ Center for Biomaterials Biomedical Research Institute Korea Institute of Science and Technology (KIST) Seoul 02792 Republic of Korea; ^4^ Department of Integrative Energy Engineering Korea University Seoul 02841 Republic of Korea; ^5^ Artificial Intelligence (AI) Graduate School Gwangju Institute of Science and Technology (GIST) Gwangju 61005 Republic of Korea; ^6^ Department of Semiconductor Engineering Gwangju Institute of Science and Technology (GIST) Gwangju 61005 Republic of Korea; ^7^ School of Electrical Engineering Korea Advanced Institute of Science and Technology (KAIST) Daejeon 34141 Republic of Korea

**Keywords:** bio‐inspired thermal adaptation, biological thermal adaptation, thermal management, thermal utilization

## Abstract

In nature, biological species have evolved unique mechanisms and sophisticated structures for intelligent radiation‐based thermal management and utilization over billions of years through natural selection. These adaptive strategies in biological species serve as a significant source of inspiration for the development of advanced thermal engineering materials and systems in modern society, driving innovation in applications such as building heating and cooling, personal thermal management, water acquisition, and next‐generation infrared (IR) sensing systems. In this review, advancements in biological and bio‐inspired thermal management strategies, including radiative cooling, thermal regulation, and thermal insulation, are comprehensively summarized. Additionally, recent advancements in radiation‐based biological and bio‐inspired thermal utilization are discussed, focusing on applications such as water harvesting, IR camouflage, and IR detection. Next, various examples of the integration of IR management strategies with electronic and energy systems are introduced, highlighting their potential to enhance efficiency and functionality in thermal management, energy efficiency, and advanced sensing applications. By leveraging these bio‐inspired systems, innovative strategies have emerged, encompassing both thermal management and utilization, and enabling efficient heat regulation and energy harvesting across a wide range of technological applications. Finally, a future perspective on the development of radiation‐based bio‐inspired thermal management and utilization technologies is provided.

## Graphical abstract

Biological organisms have evolved remarkable strategies to manage and exploit thermal radiation. This review explores bio‐inspired systems that mimic these adaptations—such as radiative cooling, thermal regulation, thermal insulation, water harvesting, infrared camouflage, and infrared detection—and highlights their application in thermal management and utilization across wearable electronics, building materials, and infrared sensing technologies.

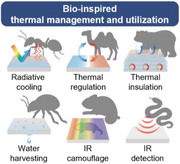

## 1 Introduction

**Figure 1:**
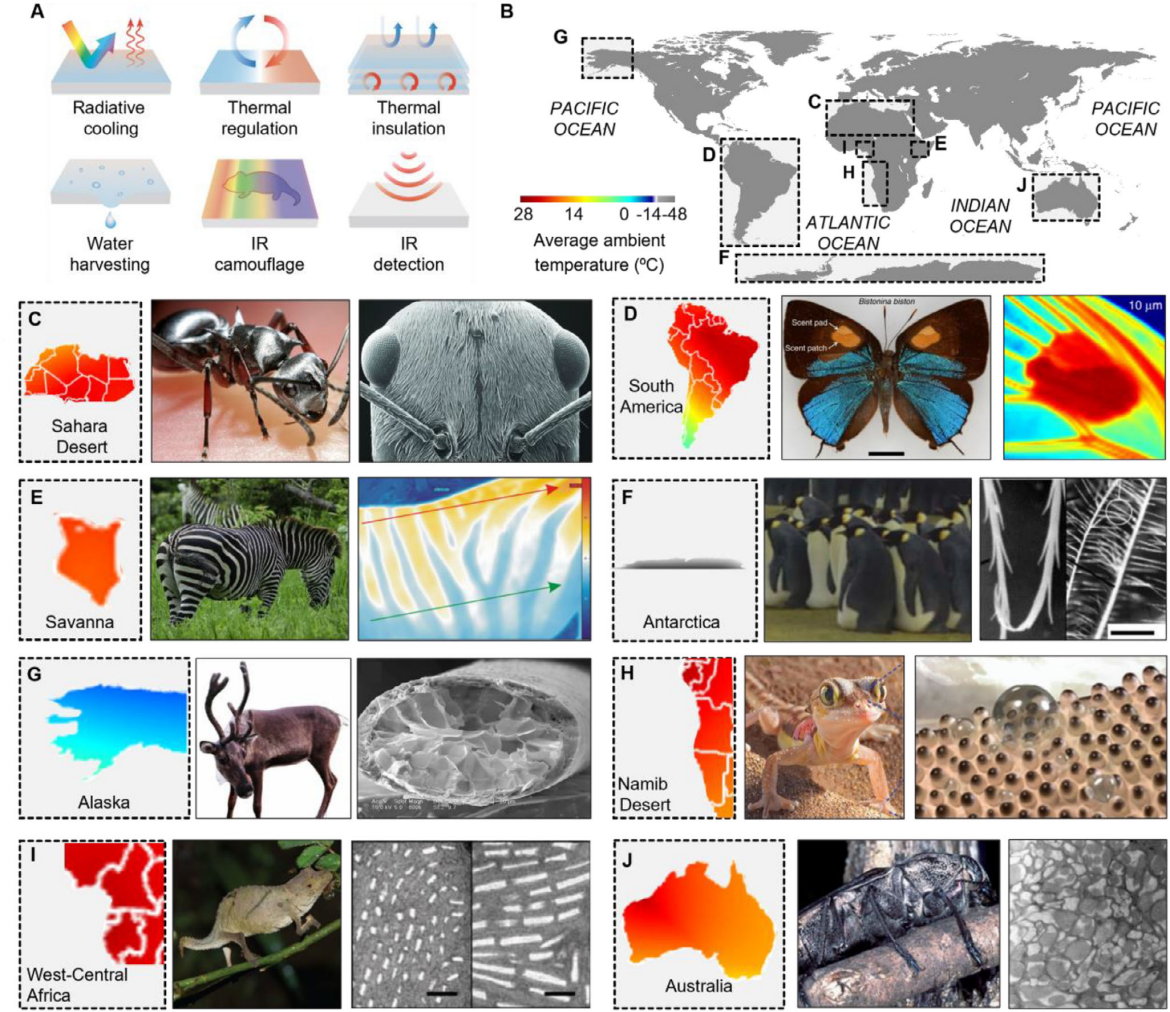
Diversity in thermal management and utilization mechanisms of organisms across different climate habitat. A) Schematic illustration of radiation‐based thermal adaptations in biological systems, illustrating various functions: radiative cooling through thermal emission, thermal regulation via controlled heat exchange, thermal insulation to minimize heat loss, water harvesting mechanisms for survival in arid environments, infrared (IR) camouflage to evade thermal detection, and IR detection for sensing environmental temperature variations. B) Global map showing average ambient temperatures across various climatic regions, highlighting C–J) thermal management and utilization strategies in biological species: (C) A Saharan silver ant (*Cataglyphis bombycina*) with triangular hairs which enable radiative cooling and minimize heat absorption, facilitating survival in extreme hot and dry Sahara Desert. Reproduced with permission.^[^ ^7^ ^]^ Copyright 2015, AAAS. Reproduced with permission.^[^ ^8^ ^]^ Copyright 2020, Elsevier. (D) A *Bistonina biston* butterfly found in South America with scent patch and scent pad on forewings, which shows high emissivity to facilitate radiative cooling. Reproduced with permission.^[^ ^9^ ^]^ Copyright 2020, Springer Nature. (E) Zebras (*Equus quagga*) living in Savanna with black and white stripe patterns to regulate body temperature through convective air eddies induced by temperature gradients. Reproduced with permission.^[^ ^12^ ^]^ Copyright 2018, Springer Nature. Reproduced with permission.^[^ ^13^ ^]^ Copyright 2020, Elsevier. (F) Emperor penguins (*Aptenodytes forsteri*) adapted to extreme cold Antarctica with down feathers consisting of aligned long rami and parallel short barbules to minimize heat loss. Reproduced with permission.^[^ ^14^ ^]^ Copyright 1999, Academic Press. Reproduced with permission.^[^ ^15^ ^]^ Copyright 2013, Royal Society. (G) A reindeer (*Rangifer Tarandus*) with porous form structure of fur to survive in extremely cold Alaska region. Reproduced with permission.^[^ ^19^ ^]^ Copyright 2019, Elsevier Ltd. (H) A Namib sand gecko (*Pachydactylus rangei*) with ellipsoidal structured on mouth and toes enable fog water collection through droplet formation in the arid Namib Desert of Southern Africa. Reproduced with permission.^[^ ^20^ ^]^ Copyright 2024, Wiley‐VCH. (I) A pygmy chameleon (*Rhampholeon spectrum*) with two superposed layers of iridophores, facilitating dynamic infrared (IR) camouflage. Reproduced with permission.^[^ ^21^ ^]^ Copyright 2022, Public Library of Science. Reproduced with permission.^[^ ^22^ ^]^ Copyright 2015, Springer Nature. (J) The Australian fire‐beetle (*Melanophila atrata*) with specialized infrared organs with unique nano architectural structures, enabling efficient detection of forest fires. Reproduced with permission.^[^ ^27^ ^]^ Copyright 2014, Emerald Group Publishing Limited.

A vast repository of survival strategies has been accumulated over billions of years within organisms inhabiting diverse ecosystems, inherited through generations from their ancestors. This extensive collection of adaptive techniques, ranging from thermal regulation to utilization, underscores the significance of habitat in understanding how organisms manage and exploit their environments. Originally derived from the classification of ecosystems by 19th‐century biogeographers such as Von Humboldt and Bonpland, the concept of habitat has since evolved. These pioneers mapped the world's major ecosystems—such as deserts and tropical rainforests—by integrating observed vegetation with climatic conditions, providing a foundation for further exploration into how organisms adapt to their surroundings.^[^ ^1^, ^2^ ^]^ In biological systems, radiation‐based strategies for thermal management and utilization have evolved to optimize thermal energy exchange with the surroundings, enhancing survival and adaptive advantages (**Figure** **1**A). Thermal management mechanisms, including radiative cooling, thermal regulation, and thermal insulation, enable organisms to regulate heat transfer efficiently, providing benefits such as thermal stability, energy conservation, and enhanced environmental adaptability.^[^ ^3^, ^4^, ^5^ ^]^ Meanwhile, thermal utilization strategies, such as water harvesting, infrared (IR) camouflage, and IR detection, exploit thermal radiation to facilitate resource acquisition, predator avoidance, and environmental awareness, thereby improving survival and ecological interactions.^[^ ^6^ ^]^

The environment inhabited by biological organisms is spread globally, from intensely cold regions such as the Antarctic and the Arctic to hot areas including deserts (Figure 1B). To adapt to these harsh and thermally extreme environmental conditions, biological species have evolved diverse strategies for thermal management, such as radiative cooling, thermal regulation, and thermal insulation. For example, the surface temperature of the Sahara Desert can reach between 60 and 70 °C.^[^ ^7^ ^]^ Inhabiting this extreme environment, the Saharan silver ants, *Cataglyphis bombycina*, have evolved triangularly structured hair that enables thermal management (Figure 1C). This unique adaptation allows the Saharan silver ants to dissipate excessive heat and minimize the absorption of solar radiation, thereby maintaining their body temperature within the operative environmental range.^[^ ^7^, ^8^ ^]^ Another example is a *Bistonina biston* butterfly inhabiting neotropical regions such as South America (Figure 1D). Tsai et al. reported that scent patches and scent pads on the forewing, which contain living cells, exhibit an average IR emissivity close to 100%, while areas without living cells have a significantly lower IR emissivity.^[^ ^9^, ^10^ ^]^ This high IR emissivity facilitates radiative cooling, maintaining the living cells at a relatively low temperature. Besides the microstructures found in the Saharan silver ants and *Bistonina biston* butterflies, the zebra (*Equus quagga*) possesses stripes for regulating body temperature (Figure 1E).^[^ ^11^, ^12^, ^13^ ^]^ These distinctive zebra stripes are composed of black stripes with strong solar absorptivity and white stripes with high solar reflectivity. The temperature gradient between two types of stripes induces convective air movements, enabling efficient thermal management under the intense solar radiation of the Savanna grasslands. This adaptation helps zebras to dissipate heat effectively and maintain a stable internal temperature despite the harsh external conditions. Additionally, for biological species living in cold environments such as the Antarctic and the Arctic, maintaining their temperature is crucial for survival. Emperor penguins, *Aptenodytes forsteri*, rely on the evenly packed feathers to minimize convection heat loss, enabling them to survive in the Antarctic with an ambient temperature as low as −40 °C (Figure 1F).^[^ ^14^, ^15^, ^16^ ^]^ In environments as extreme as the Arctic and Greenland, similar to Alaska,^[^ ^17^ ^]^ the reindeer (*Rangifer tarandus*) has evolved fur microstructures adapted to these harsh conditions (Figure 1G).^[^ ^18^, ^19^ ^]^ The sophisticated porous foam structure of the reindeer's fur reduces convective heat loss similar to the feathers of the emperor penguin and demonstrates significant thermal insulating capabilities in extremely cold environments. This adaptation allows reindeer to maintain a warm body temperature.

Beyond the thermal management capabilities of organisms, biological species also utilize thermal radiation in extreme environments for various survival strategies, such as water harvesting, IR camouflage, and IR detection. The Namib sand gecko (*Pachydactylus rangei*) facilitates water harvesting in the arid environment of the Namib Desert through its low skin temperature and ellipsoidal structured skin (Figure 1H).^[^ ^20^ ^]^ Specifically, the Namib sand gecko buries itself within the sand to evade thermal radiation and reduce its body temperature. Before dawn, it ascends a dune to condense moisture on its skin, which features ellipsoidal structures, thereby facilitating moisture harvesting. This unique survival strategy enables the Namib sand gecko to efficiently acquire water in areas with limited water availability. Biological species also possess advanced capabilities in both visible and IR camouflage, allowing them to disguise themselves in the background from predators, even those equipped with IR vision. For example, the pygmy chameleon (*Rhampholeon spectrum*), predominantly found in West‐Central Africa,^[^ ^21^ ^]^ features a skin composed of two layers of iridophores: superficial and deep iridophore layers (Figure 1I).^[^ ^22^ ^]^ Each layer plays a distinct role in camouflage across different spectra, including visible and IR wavelengths. The lattice of guanine nanocrystals within superficial iridophore layers is actively tuned to facilitate visible camouflage. Meanwhile, the larger crystalline structures in the deep iridophores effectively reflect infrared light, extending their camouflage capabilities into the IR wavelength. This dual‐layer structure of the pygmy chameleon's skin allows it to adaptively alter its appearance for concealment. Moreover, certain animals, such as rattlesnakes, exhibit the capability to remotely detect IR radiation emitted from their surroundings for mating, tracking prey, and searching for shelters.^[^ ^23^, ^24^, ^25^ ^]^ The fire beetle (*Melanophila atrata*) is a representative biological species with outstanding IR detection capabilities for locating shelters (Figure 1J).^[^ ^26^, ^27^ ^]^ The larvae of fire beetles rely on freshly burnt wood for habitation. Moreover, the vicinity of fire serves as a crucial mating site for breeding. Therefore, these beetles approach forest fires from considerable distances using a pair of IR pit organs located on the metathorax adjacent to the coxae of the middle legs. Each organ consists of 70 IR sensilla, which have evolved from cuticular hair mechanoreceptors. These sensilla operate through a photomechanical mechanism, in which incoming IR radiation is absorbed by the cuticular structure of the sensillum, forming a small sphere. The expansion of this sphere is then precisely measured by the dendrite of a ciliary mechanoreceptive sensory cell. This IR detection mechanism in fire‐tracking beetles facilitates survival by enabling them to search for breeding sites and shelters.

Building on the principles of natural selection and habitat adaptation introduced by Darwin and Wallace, subsequent research has enhanced our understanding of how organisms adapt to and survive in varying environmental conditions. However, while many studies have been inspired by specific interactions between climate and habitat, a comprehensive overview that synthesizes these findings is still lacking. In this review, we aim to explore the broader implications of habitat characteristics for biological strategies in thermal management and utilization. By examining how organisms regulate heat and utilize radiation within their ecosystems, we seek to bridge the knowledge gap between individual species studies and a broader understanding of radiation‐based thermal adaptation. We first investigate the biological thermal management strategies through radiative cooling, thermal regulation, and thermal insulation that enable organisms to survive in extreme environments, e.g., deserts and polar regions, to understand structural and optical features underlying these adaptive mechanisms. Then, we discuss radiation‐based bio‐inspired thermal management materials and systems to replicate the structural and optical features of natural thermal management systems. Subsequently, we explore radiation‐based thermal utilization, analyzing how strategies such as water harvesting, IR camouflage, and IR detection are facilitated by intricate biological structures and specialized natural mechanisms. We also show the design principles and performance of advanced bio‐inspired materials and systems for thermal utilization. Additionally, we investigate the integration of bio‐inspired thermal management and utilization technologies with energy and electronic systems to enhance performance and expand their application. This interdisciplinary approach offers new opportunities to overcome the limitations of conventional technologies, enabling advancements in energy‐efficient thermal management, adaptive thermal textiles, environmentally sustainable materials, and next‐generation sensing technologies. Finally, we provide a forward‐looking perspective on the future development of radiation‐based bio‐inspired thermal management and utilization technologies. Acronyms and full names in this paper are summarized in **Table** **1**.

**Table 1:** Summary of the acronyms and full names.

Acronym	Full name	Acronym	Full name
Ag	Silver	P(VDF‐HFP)	Poly(vinylidene fluoride‐co‐hexafluoropropene)
Al	Aluminum	PBS	Poly(butylene succinate)
Al_2_O_3_ NPs	Aluminum oxide nanoparticles	PCL	Polycaprolactone
ATW	Atmospheric transparent window	PCM	Phase change material
BE	Broadband emitter	PDA	Polydopamine
Bio‐PC	Bio‐inspired photonic composite	PDMS	Polydimethylsiloxane
Bio‐RC	Bio‐inspired radiative cooling	PEDOT:PSS	Poly(3,4‐ethylenedioxythiophene):poly(styrene sulfonate)
Bi‐Te	Bismuth telluride	PGS	Poly(glycerol sebacate)
ChR2	Channelrhodopsin‐2	PGCL	Poly(glycolide‐co‐ε‐caprolactone)
CNT	Carbon nanotube	PI	Polyimide
CP	Chromatophore	PLA	Polylactic acid
CPMn	Carbon nanotube/polylactic acid/manganese dioxide	PLCL	Poly(lactide‐ε‐caprolactone)
CPV	Concentrating photovoltaic	PMMA	Polymethylmethacrylate
DBTDL	Dibutyltin dilaurate	PPG	Photoplethysmography
EAF	Encapsulated aerogel fiber	PVDF	Poly(vinylidene fluoride)
EGaIn	Eutectic gallium‐indium	P(VDF‐HFP)	Poly(vinylidene fluoride‐co‐hexafluoropropylene)
EMPA	Electrospun micro‐pyramid array	Si NM	Silicon nanomembrane
FIR	Far‐infrared	SiO_2_	Silicon dioxide
FPA	Flexible photonic architectures	S‐SLO	Shell‐like superhydrophilic origami
HDI	Hexamethylene diisocyanate	TiO_2_	Titanium dioxide
IP	Iridophore	TPU	Thermoplastic polyurethane
IR	Infrared	TRP	Transient receptor potential
LP	Leucophore	UCNP	Upconversion nanoparticle
LWIR	Long‐wave infrared	USRI	Ultrathin, soft, radiative cooling interface
MCB	Micro‐bar‐array	UV	Ultraviolet
MEPFT	Micro‐extruded physically foamed porous elastic fiber with TPU elastomer	vis	Visible
MEPFT‐d	Double‐layered fabric using MEPFT	V_OC_	Open‐circuit voltage
MgHPO_4_	Magnesium phosphate hydrate	W	Tungsten
MIR	Mid‐infrared	ZnO	Zinc oxide
MnO_2_	Manganese dioxide	ZrC	Zirconium carbide
NIR	Near‐infrared		

### 1.1 Bio‐Inspired Thermal Management and Utilization Systems Inspired by Nature

**Figure 2:**
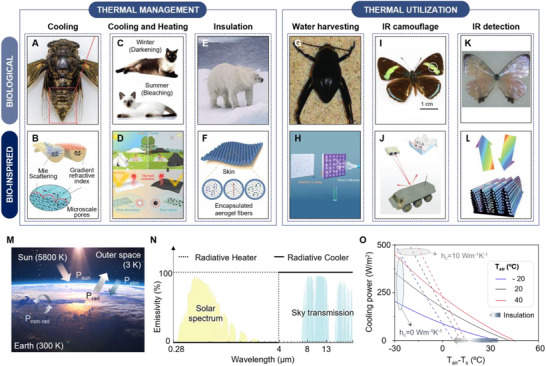
Overview of biological and bioinspired thermal management and thermal utilization. A) Optical image of a cicada (*Cryptotympana atrata*) with porous heart shaped structure. B) Illustration of Bio‐PC for radiative cooling, composed of TPU embedded with Al_2_O_3_ NPs. Reproduced with permission.^[^ ^28^ ^]^ Copyright 2021, Wiley‐VCH. C) Photograph of a Siamese cat, *Felis catus*, showing thermally induced color changes. D) Schematic illustration of reversible thermochromic fiber membrane with temperature‐adaptive dual‐mode thermal management performance utilizing thermochromic microencapsulated phase change material. Reproduced with permission.^[^ ^29^ ^]^ Copyright 2024, American Chemical Society. E) Photograph of a polar bear, *Ursus maritimus*. Reproduced with permission.^[^ ^31^ ^]^ Copyright 2025, AAAS. F) Schematic of an EAF for thermal insulation. Reproduced with permission.^[^ ^32^ ^]^ Copyright 2023, AAAS. G) Image of a Namib desert beetle (*Onymacris unguicularis*) with bump microstructures on elytra for fog collection. Reproduced with permission.^[^ ^33^ ^]^ Copyright 2010, Springer Nature. H) Schematic diagram of water harvesting device with P(VDF‐HFP) cooling coating embedded with silicon dioxide (SiO_2_) and Calcium molybdate (CaMoO_4_) nanoparticles for efficient water harvesting, mimicking beetle elytra structures. Reproduced with permission.^[^ ^34^ ^]^ Copyright 2020, Wiley‐VCH. I) Photographs of *Diaethria clymena* butterflies with green scales, showing camouflage and reflection splitting properties. J) Schematic of bioinspired meta‐reflection‐splitter utilizing aluminum (Al)‐polydimethylsiloxane (PDMS) based metasurface for thermal camouflage. Reproduced with permission.^[^ ^36^ ^]^ Copyright 2023, Wiley‐VCH. K) Optical image of *Morpho sulkowski* butterfly with a fractured scale on the wing. L) Illustration of IR detector utilizing *Morpho* scales structures deposited single‐walled carbon nanotube, resulting in wavelength conversion. Reproduced with permission.^[^ ^37^ ^]^ Copyright 2012, Springer Nature. M) Schematic illustration of radiative heat transfer occurring at terrestrial surfaces. *P*_sun_, absorbed power from sunlight; *P*_rad_, radiated power by the emitter; *P*_atm_, absorbed power from the atmosphere; *P*_non‐rad_, non‐radiative heat transfer coefficient. N) Solar spectrum (yellow shaded area) and the sky transmission spectrum (blue shaded area) in the mid‐infrared wavelength range. The solid and dashed line represent the ideal emissivity spectra of the radiative heater and cooler, respectively. O) Net cooling power as the function of the difference between temperature of ambient air (*T*_air_) and sample (*T*_s_). The blue, black, and red correspond to *T*_air_ of −20, 20, and 40 °C, respectively. The solid and dashed lines indicate non‐radiative heat transfer coefficients (*h*_c_) of ≈0 and 10 Wm^−1^K^−1^, respectively.

Bio‐inspired engineering takes advantage of the unique mechanisms and delicate structures in biological systems through billions of years of natural selection. These biological structures serve as profound sources of inspiration for the development of sophisticated thermal engineering materials and systems with exceptional performance. Consequently, there have been numerous efforts to develop thermal management and utilization techniques by using bio‐inspired approaches that replicate the unique survival strategies in biological species. For example, the golden microspike on cicada, *Cryptotympana atrata*, with a porous heart‐shaped cross‐section shows radiative cooling properties that avoid overheating in the hot summer (**Figure** **2**A).^[^ ^28^ ^]^ Liu et al. fabricated a bio‐inspired photonic composite (Bio‐PC) consisting of thermoplastic polyurethane (TPU) and aluminum oxide nanoparticles (Al_2_O_3_ NPs) by mimicking the unique biological structure (Figure 2B). The nanopores within the Bio‐PC structure enhance reflectivity in the visible (vis) to near‐infrared (NIR) range to 97.6% through Mie scattering, and the fluctuant surfaces lead to high IR emissivity of 95.5% owing to graded‐index interface, resulting in enhanced radiative cooling performance. For organisms living in environments with large diurnal temperature variations or seasonal temperature changes, thermal regulation is crucial for their survival through both cooling and heating functionalities. Siamese cat (*Felis catus*) has temperature‐sensitive hair that changes color in response to seasonal temperature variations (Figure 2C).^[^ ^29^ ^]^ The dynamic color change of hair in Siamese cats adjusts solar‐thermal energy conversion efficiency to maintain their body temperature within a comfortable range. The temperature‐sensitive catalysis from Siamese cats has inspired the fabrication of a novel temperature‐adaptive thermal management membrane using poly(vinylidene fluoride‐co‐hexafluoropropene) (P(VDF‐HFP)) and thermochromic encapsulated phase change materials (Figure 2D). During the summer, when the ambient temperature is 38 °C, the temperature‐adaptive thermal management membrane shows a lower temperature of 3 °C compared to ambient temperature, providing an effective cooling performance. In contrast, during the winter, when the ambient temperature is 20 °C, the membrane maintains a higher temperature of 2 °C than that of ambient air, demonstrating its heating capability. Additionally, maintaining or increasing body temperature is crucial for the survival of organisms living in cold environments. For example, the unique structure of polar bear hairs reduces heat loss from their bodies to the environments which enables them to keep warm for their survival (Figure 2E).^[^ ^16^, ^30^, ^31^ ^]^ Inspired by these outstanding biological thermal insulation characteristics, Wu et al. developed an encapsulated aerogel fiber (EAF) for thermal insulation textiles using TPU, which shows high stretchability and mechanical strength (Figure 2F).^[^ ^32^ ^]^ The EAF has significant potential in thermal apparel because it is knittable owing to the flexibility and stretchability of TPU. In extremely cold conditions of −20 °C, the EAF sweater shows a surface temperature of 3.5 °C, outperforming the thermal insulation performance of traditional wool (7.2 °C) and cotton sweaters (10.8 °C). This lower surface temperature of EAF sweater indicates reduced heat loss from the body to the cold environments, demonstrating superior thermal insulation performance. This suggests that EAF offers effective insulation, making it ideal for lightweight winter clothing.

Besides the bio‐inspired materials for advanced thermal regulation, another crucial area of bio‐inspired research is the development of materials for next‐generation thermal utilization, including water harvesting, IR camouflage, and IR detection. To survive in the extreme aridity of desert environments, some biological species have evolved specialized adaptations that enable them to collect water from the moist air. A notable example is the Namib Desert beetle (*Onymacris unguicularis*), which possesses a bumpy surface characterized by periodic hydrophobic, wax‐coated regions and hydrophilic, non‐waxy regions (Figure 2G).^[^ ^33^ ^]^ Furthermore, the wax‐coated regions of the bumpy surface exhibit high IR emissivity, enabling radiative cooling to lower surface temperature, thereby enhancing water collection efficiency. The outstanding water harvesting capability has propelled the fabrication of a water harvesting device with a radiative cooling layer (Figure 2H).^[^ ^34^ ^]^ This integration of bio‐inspired wettability patterns and radiative cooling layer promotes efficient droplet nucleation on hydrophilic regions while facilitating rapid water transport across hydrophobic regions. Additionally, the radiative cooling coating layer dissipates latent heat released during condensation, further enhancing water collection efficiency by up to 52% compared to a water harvesting device without radiative cooling coating layer. Biological species also exhibit advanced IR camouflage capability that enhances predatory efficiency and reduces predation risks, significantly increasing the chance of survival.^[^ ^35^ ^]^ Some butterflies, such as *Diaethria clymena* and *Morpho sulkowskyi* butterflies, exhibit an IR camouflage strategy through a reflection‐splitting effect of their green scales, which leads to a 180° phase difference in asymmetrical multilayered laminate. This structural adaptation suppresses specular reflection and scatters incident light across wider angles, effectively reducing observability to aerial predators in their natural habitat.^[^ ^36^ ^]^ Inspired by the reflection‐splitting effect on the green scale of the *Diaethria clymena* butterfly, Liu et al. developed a bio‐inspired meta‐reflection‐splitter for ultralow specular reflectance and large scattering angles (Figure 2J).^[^ ^36^ ^]^ The bio‐inspired meta‐reflection‐splitter not only exhibits a scattering angle of ≈50° under the 1064 nm laser but also demonstrates low IR emissivity, thereby significantly enhancing both NIR laser and thermal stealth performance. Additionally, the wings of the *Morpho sulkowskyi* butterfly have been widely investigated for IR detection owing to their distinctive structural and optical sensitivity, combined with extremely low thermal mass (Figure 2K).^[^ ^37^ ^]^ The scales of *Morpho sulkowskyi* butterfly have an air‐filled nanoarchitecture consisting of longitudinal ridges and lamellae, allowing them to selectively reflect visible light. Taking inspiration from the air‐filled nanoarchitecture of *Morpho sulkowskyi* butterfly scales, Pris et al. reported the application of Morpho butterfly wings for IR detection (Figure 2L).^[^ ^37^ ^]^ This bio‐inspired design achieves high sensitivity, fast response, and high spatial resolution without the need for a heat sink.

### 1.2 Principles and Theoretical Model

While most living organisms on Earth maintain a body temperature of ≈300 K, their thermal regulation strategies vary depending on environmental conditions to ensure survival and normal physiological function. The thermal radiation emitted from the surface of the biological organisms can escape into outer space through the atmospheric transparent window (ATW), where the atmosphere exhibits high transparency. In contrast, for organisms inhabiting cold environments, conserving heat is critical for their survival. These species have evolved specialized biological structures that minimize heat loss by reducing thermal transfer through conduction, convection, and radiation. These adaptations enhance thermal insulation, allowing organisms to maintain stable body temperatures in extremely cold conditions. Drawing inspiration from these biological thermal management strategies, thermally engineered emitters are designed to achieve optimal performance for target applications. In many cases, polymer‐based materials are utilized to tailor the spectral emissivity of emitters, owing to their tunable chemical composition and molecular vibrational modes.^[^ ^38^ ^]^ As shown in Figure 2M, for an emitter at temperature *T* with spectral and angular emissivity *ε*(λ,  θ), the net radiative power *P*_net_(*T*) is determined by four power components and is expressed as follows:^[^ ^39^, ^40^, ^41^, ^42^ ^]^

(1) $$P_{\mathrm{net}}\left(T\right)=P_{\mathrm{rad}}\left(T\right)-\;P_{\mathrm{atm}}\left(T_{\mathrm{air}}\right)-\;P_{\mathrm{sun}}-\;P_{\mathrm{non}-\mathrm{rad}}$$

where *P*_rad_(*T*) denotes the radiated power by the emitter, *P*_atm_(*T*_air_) refers to the absorbed power from the atmosphere, *P*_sun_ is the absorbed power from sunlight, and *P*_non − rad_ refers to the heat transfer by convection and conduction between the emitter and atmosphere. *T*_air_ is the temperature of ambient air. When *P*_net_ is zero, the emitter is in a steady‐state. At steady‐state, if the temperature of the emitter is higher than that of ambient air (i.e., *T* > *T*_air_), the emitter is in a heating mode. Conversely, if the temperature of the emitter is lower than that of ambient air (i.e., *T* < *T*_air_), the emitter is in a cooling mode. The four power terms are expressed as follows:^[^ ^39^, ^40^, ^41^, ^42^ ^]^

(2) $$P_{\mathrm{rad}}\left(T\right)=\int_{0}^{2\pi }\int_{0}^{\pi /2}\int_{0}^{\infty }I_{\mathrm{BB}}\left(T,\lambda \right)\varepsilon \left(\lambda ,\theta \right)\cos\left(\theta \right)\sin\left(\theta \right)d\lambda d\theta d\phi$$

(3) $$\begin{array}{ccc} & & P_{\mathrm{atm}}\left(T_{\mathrm{air}}\right)=\\ & & \int_{0}^{2\pi }\int_{0}^{\pi /2}\int_{0}^{\infty }I_{\mathrm{BB}}\left(T_{\mathrm{air}},\lambda \right)\varepsilon \left(\lambda ,\theta \right)\varepsilon_{\mathrm{amb}}\left(\lambda ,\theta \right)\cos\left(\theta \right)\sin\left(\theta \right)d\lambda d\theta d\phi \end{array}$$

(4) $$P_{\mathrm{sun}}=\int_{0}^{\infty }I_{\mathrm{AM}1.5G}\left(\lambda \right)\varepsilon \left(\lambda \right)d\lambda$$

(5) $$P_{\mathrm{non}-\mathrm{rad}}=\left(T-T_{\mathrm{air}}\right)h_{c}$$

where $I_{\mathrm{BB}}\left(T,\lambda \right)=\left(2hc^{2}/\lambda^{5}\right)/\left[e^{hc/\lambda k_{B}T}-1\right]$ denotes the spectral radiance of a blackbody at temperature *T*, and *h*, *c*, *k*_B_, *λ*, and *h*_c_ refer to the Planck's constant, velocity of the light, the Boltzmann constant, wavelength, and non‐radiative heat coefficient, respectively. *ε*_amb_(*λ*, *θ*) = 1 − *t*(*λ*)^1/cos (*θ*)^ is the atmospheric emissivity, where *t*(*λ*) is the atmospheric transmittance in the zenith direction.

For thermal emitters, precise spectral engineering is essential to optimize their radiative properties and implement an appropriate strategy, such as a radiative cooler or heater, that is suitable for target applications. As shown in Figure 2N, the performance of a thermal emitter is strongly influenced by specific wavelength regions, such as the solar spectrum (0.28–4 µm) and the IR wavelength range. The solar spectrum consists of distinct wavelength ranges, including ultraviolet (UV; 0.28–0.4 µm), vis (0.4–0.8 µm), NIR (0.8–1.1 µm), and mid‐infrared (MIR; 1.1–4 µm), which accounts for the majority of incident solar radiation. The IR wavelength is generally classified into the long‐wave infrared (LWIR; 4–13 µm) and the far‐infrared (FIR) region, which encompasses wavelengths beyond 13 µm. Notably, the 8–13 µm wavelength range within the LWIR range is known as the ATW, where the atmosphere exhibits high transparency, enabling efficient thermal radiation from the surface of the emitter to escape into outer space. Optimizing the optical characteristics (i.e., reflection, transmission, and absorption) across specific wavelength regions enables the design of materials with radiative cooling or heating properties for distinct applications. For radiative cooling, materials with high reflection within the entire solar spectrum minimize solar absorption, while strong absorption in the LWIR and FIR regions enables efficient heat dissipation into outer space (Figure 2M; solid line). In contrast, radiative heating applications require materials that efficiently absorb solar energy while reducing thermal emission in the LWIR and FIR regions to maintain heat (Figure 2M; dashed line). Under real‐world conditions, the thermal performance of an emitter is dictated by the interplay between radiative and non‐radiative heat transfer. Figure 2O illustrates the equilibrium temperature of an ideal broadband emitter (BE) as a function of ambient temperature (*T*_air_). The ideal BE is characterized by zero solar absorption within the 0.25–4 µm wavelength range and 100% thermal emission in the 4–30 µm range. The intersection of each curve with the *x*‐axis (i.e., the cooling power reaches zero) represents the temperature difference between the sample and ambient air (*T*_air_ − *T*_s_), referred to as the cooling temperature. To simplify the calculation, thermal conduction is assumed to be negligible and only convective heat transfer is considered. Accordingly, *h*_c_ represents the convection heat transfer coefficient, where *h*_c_ = 0 Wm^−1^K^−1^ corresponds to a no‐wind condition, and *h*_c_ = 10 Wm^−1^K^−1^ represents a scenario with a constant wind speed of 1 ms^−1^.^[^ ^43^ ^]^ As shown in Figure 2O, higher *T*_air_ leads to superior cooling performance, regardless of wind speed (i.e., a higher *T*_air_ results in a higher cooling temperature). Interestingly, the effect of convection (*h*_c_ = 10 Wm^−1^K^−1^) on ideal BE does not always enhance the cooling performance. Compared to a no‐wind condition (*h*_c_ = 0 Wm^−1^K^−1^), minimizing convective heat loss allows the BE to function similarly to thermal insulation. This suggests that restricting convective heat transfer enhances thermal insulation, indicating the potential for reducing overall heat dissipation.

## 2 Radiative‐Based Thermal Management in Biological and Bio‐Inspired System

### 2.1 Radiative Cooling

Biological organisms in extremely hot environments have evolved elaborate surface microstructures to efficiently dissipate thermal energy, which has led to the development of bio‐inspired radiative coolers for contributing to energy saving in human society. In this section, we first investigate recent developments in bio‐inspired radiative coolers, focusing on key replication strategies, optical properties, and distinctive working principles. Additionally, we introduce microstructures of insects that have not yet been emulated, providing novel inspirations for the advancement of innovative bio‐inspired radiative coolers.

#### 2.1.1 Triangular prismatic structure inspired by Saharan silver ants

**Figure 3:**
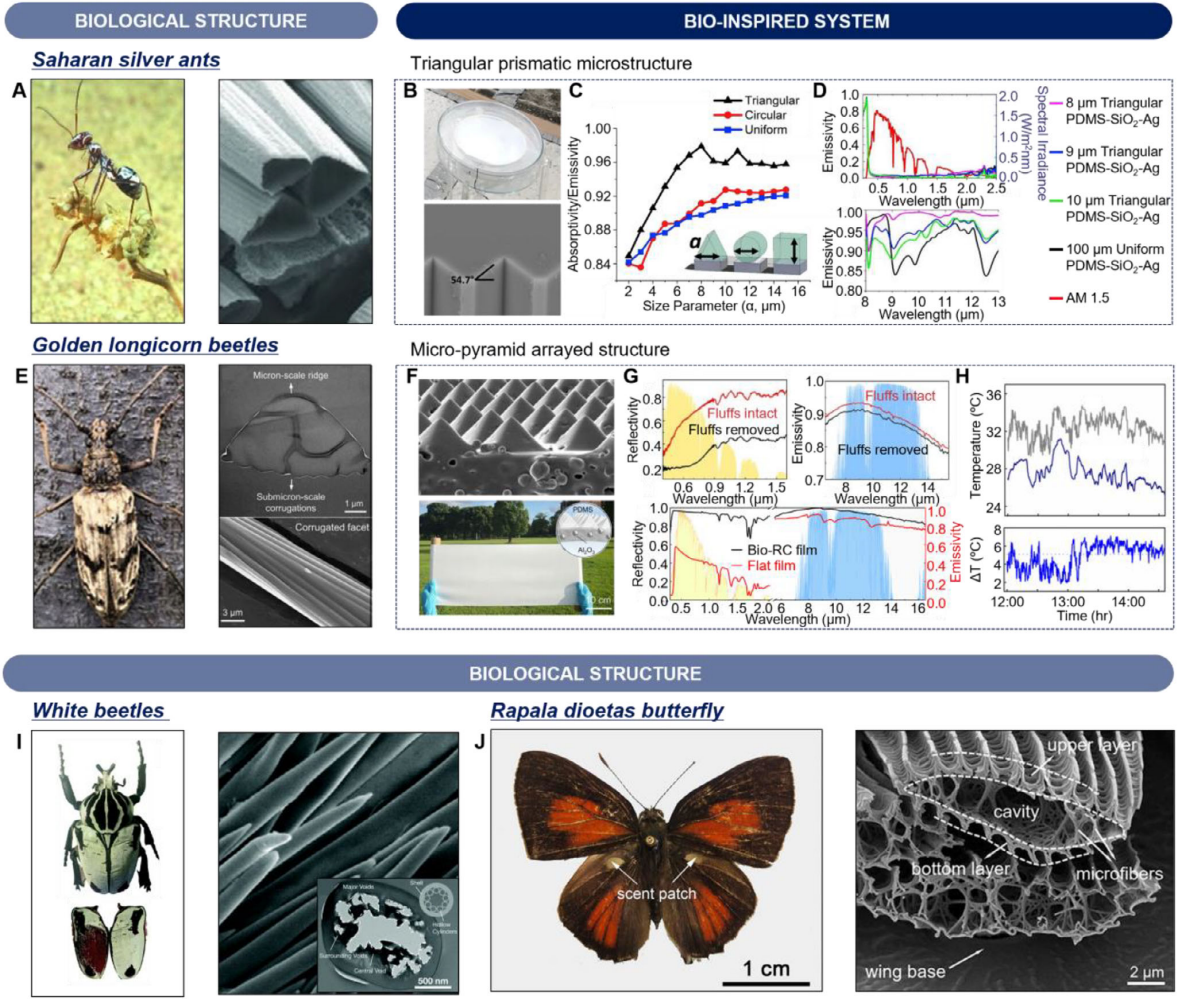
Biological organisms and bio‐inspired materials for radiative cooling. A) Optical image of a Saharan silver ant (*Cataglyphis bombycina*) (left) and scanning electron microscope (SEM) image (right) of the cross‐sectional view of hairs on silver ant, presenting a dense layer of triangularly shaped hairs. Reproduced with permission.^[^ ^7^ ^]^ Copyright 2015, AAAS. B) Photograph image of fabricated triangularly shaped PDMS‐SiO_2_‐Ag three layered radiative cooler (top) and SEM image of patterned silicon mold. C) Simulation results of averaged emissivity in the ATW of different geometrical structures (triangular, circular, and uniform arrays) with size parameter of 2–15 µm. D) Measured results of emissivity spectra of the 100 µm‐thick uniform PDMS‐SiO_2_‐Ag cooler (black line) and the patterned PDMS‐SiO_2_‐Ag cooler featuring a triangular prism array with a size parameter of 8 µm (pink line), 9 µm (blue line), and 10 µm (green line) in the wavelength range from 0.25 µm to 2.5 µm and 8 µm to 13 µm (top and bottom, respectively). The red line indicates AM1.5 spectrum. Reproduced with permission.^[^ ^8^ ^]^ Copyright 2019, Elsevier. E) Photograph of a male golden longicorn beetle (*Neocerambyx gigas*) (left), cross‐sectional transmission electron microscope (TEM) of a fluff on the forewing (top right), and SEM image of corrugated facet of the fluff (bottom right). F) SEM image of micro‐pyramid arrayed PDMS polymer matrix containing randomly distributed Al_2_O_3_ spheres (top) and photograph of Bio‐RC film (bottom). G) Spectral characteristics of fluffs of golden longicorn beetles (top) and a 500‐µm‐thick Bio‐RC film with randomly distributed 100‐nm‐diameter Al_2_O_3_ particles. In visible wavelength range, the Bio‐RC film shows high reflectivity owing to total internal reflection and Mie scattering. The strong phonon–polariton resonances induced by Al_2_O_3_ particles and gradual refractive index of pyramid shape, leading to increased IR emissivity. H) Measured temperature of the air (gray line) and Bio‐RC film (blue line) (top) and temperature difference between air and Bio‐RC film (bottom). Reproduced with permission.^[^ ^47^ ^]^ Copyright 2020, PNAS. I) Photograph of a male white beetle (*Goliathus goliatus*) and SEM image of the central part of white scales. Inset: cross‐sectional TEM image of the white scales with exterior shell and interior of packed hollow cylinders, resulting in broadband omnidirectional reflection such as thin‐film interference, Mie scattering, and total internal reflection. Reproduced with permission.^[^ ^51^ ^]^ Copyright 2019, Royal Society of Chemistry. J) Photograph of a male *Rapala dioetas* butterfly (left) and cross‐sectional SEM image (right) of scent patch scales on the hindwing, featuring high IR emissivity due to hierarchical microstructure. Reproduced with permission.^[^ ^52^ ^]^ Copyright 2021, AIP Publishing.

In extremely hot environments, such as deserts, some insects have evolved thermal regulation strategies to reduce their body temperatures by minimizing the absorption of solar radiation and enhancing IR radiation. For example, the Saharan silver ants, *Cataglyphis bombycina*, inhabit one of the harshest climates on Earth, the Sahara Desert. To thrive in such harsh environments, Saharan silver ants are required to dissipate excessive heat and suppress the heat gain from the environment. Wehner et al. revealed that the dorsal and lateral sides of these ants are covered by dense, uniform arrays of hairs and show silvery glare (**Figure** **3**A; left).^[^ ^7^ ^]^ The structural feature of these hairs is a triangular cross‐section, featuring two corrugated top facets and a flat bottom facet facing the epidermis (Figure 3A; right). This configuration serves as a gradient refractive index layer, offering the surface with broadband IR emissivity. Additionally, enhanced solar reflectivity is achieved due to Mie scattering and internal reflection by the hair structure of Saharan silver ants.

These thermal management strategies of Saharan silver ants have inspired the development of bio‐inspired coatings for passive radiative cooling.^[^ ^8^, ^44^, ^45^, ^46^ ^]^ For example, Jeong et al. fabricated a prismatic polymer‐based passive radiative cooler (triangular‐shaped polydimethylsiloxane (PDMS)‐SiO_2_‐silver (Ag) three‐layered radiative cooler) (Figure 3B; top).^[^ ^8^ ^]^ The structure of the triangular shape of PDMS was designed by using silicon mold (Figure 3B; bottom). To investigate the impact of the triangular shape on thermal emission properties, the IR emissivity of three geometrical modifications—triangular, circular, and uniform configurations—was analyzed using finite difference time domain simulations (Figure 3C). The triangular configuration shows the highest value compared to the other two configurations (i.e., circular and uniform configurations) owing to a gradient refractive index change between air and PDMS in the LWIR wavelength range. As shown in Figure 3D, the emissivity spectra in the UV–NIR (Figure 3D; top) and LWIR (Figure 3D; bottom) regions of the coolers with PDMS in triangular configurations demonstrated an enhanced average emissivity of 98% in the IR wavelength range, with an average reflectivity of 95% in the vis–NIR spectrum. The optimized PDMS‐SiO_2_‐Ag passive radiative cooler exhibits a lower average temperature of 6.2 °C compared to that of ambient air in a tropical climate.

Furthermore, Lin et al. fabricated flexible photonic architectures (FPA)‐PDMS by exploiting silicon masters to mimic corrugated triangular hairs of Saharan silver ants.^[^ ^44^ ^]^ The fabricated FPA‐PDMS structure achieves LWIR emissivity of 98% and vis–NIR transmittance of 97%, which makes it attractive for photovoltaic devices to maximize sunlight absorption and reduce parasitic heat. Also, a triangular air gap was fabricated on PDMS, enhancing solar reflectivity and IR emissivity by 17% and 4%, respectively, showing an average cooling temperature of 5.6 °C.^[^ ^45^ ^]^ Additionally, a novel solar heat shielding fabric was suggested using a zinc oxide (ZnO) micro‐crystal‐bar composite, achieving solar reflectivity of 95% and a maximum cooling temperature of 11.7 °C than other samples.^[^ ^46^ ^]^ The passive radiative coolers inspired by the triangular structure of silver ants are fabricated using various materials and methods. Additionally, this innovation not only offers promising applications in thermal management but also shows potential for enhancing the efficiency of photovoltaic devices.

#### 2.1.2 Micro‐pyramid arrayed structure inspired by golden longicorn beetles

Similar to the Saharan silver ants, golden longicorn beetles, *Neocerambyx gigas*, possess triangular cross‐section microstructures with dual‐scale triangular fluffs that have thermal regulation abilities in hot regions.^[^ ^47^ ^]^ As shown in Figure 3E (left), the golden longicorn beetles exhibit a golden glare owing to fluffs on the elytra of forewings. The fluffs exhibit a triangular shape, forming a dual‐scale structure with two flat facets and one corrugated facet facing the epidermis (Figure 3E; right). The high reflectivity of the forewings is due to the combined effects of Mie scattering at the edges of the fluffs and total internal reflection within the triangular structure.

Taking inspiration from the distinctive features of the golden longicorn beetles, a flexible hybrid photonic film was designed for passive radiative cooling.^[^ ^47^ ^]^ The bio‐inspired radiative cooling (Bio‐RC) film is composed of a periodic micro‐pyramid array of PDMS that encapsulates randomly dispersed spherical ceramic particles, such as titanium dioxide (TiO_2_), Al_2_O_3_, and ZnO (Figure 3F). Due to the strong absorption induced by resonant polar dielectric microspheres with a gradual refractive index of micro‐pyramid arrays, the Bio‐RC film exhibits high emissivity in the LWIR wavelength range. Simultaneously, the effective Mie scattering of the encapsulated particles and the total internal reflection within the triangular structure in the vis–NIR range, the Bio‐RC film demonstrates exceptionally high solar reflectivity. In the vis–NIR spectrum, the fluff‐coated regions exhibit an average solar reflectivity of 65%, which is 35% higher than that of the fluff‐removed regions (Figure 3G; top left). Furthermore, the fluff‐coated regions on the forewings demonstrate peak emissivity of 94%, effectively radiating body heat into the environment. Similar to the optical properties of the fluff on the elytra, the Bio‐RC film exhibits solar reflectivity of 95% and LWIR emissivity of 96% (Figure 3G; bottom). These spectral properties enable superior sub‐ambient cooling performance with an average cooling temperature of 5.1 °C and maximum cooling temperature of 7 °C, showing an average cooling power of 90.8 Wm^−2^ (Figure 3H). Moreover, when combined with its hydrophobic nature, exceptional flexibility, and robust mechanical strength, the film shows immense potential for use in wearable thermal management devices.

Further research has been conducted on the radiative cooling abilities of polymer‐based micro‐pyramid structure, which provides superior selective emissivity in the LWIR wavelength and reflectivity in the solar spectrum. Zhang et al. reported an electrospun micro‐pyramid array (EMPA) fabric based on poly(vinylidene fluoride) (PVDF).^[^ ^48^ ^]^ The EMPA fabric shows high vis–NIR reflectivity of 97.9% and LWIR emissivity of 76.3%, providing an average cooling temperature of 4 °C under 1‐sun conditions. Similarly, PDMS films with a polymer‐based microphotonic multifunctional metamaterial surface were prepared as a radiative cooling film, achieving LWIR emissivity of 98%. This film exhibits excellent radiative cooling performance with an average cooling temperature of 6 °C than ambient air.^[^ ^49^ ^]^ Additionally, a random inverted pyramid‐like light‐trapping structure can be implemented on the surface of the PDMS film through cost‐effective coating processes, which enhances cooling performance by 2.7%, making it more suitable for use as an outdoor radiative cooling emitter.^[^ ^50^ ^]^

#### 2.1.3 Microstructure of white beetles and Rapala dioetas butterfly for radiative cooling

In nature, diverse biological species have evolved sophisticated structural adaptations that enable them to efficiently regulate thermal radiation, allowing them to survive even in extremely hot conditions. These adaptations include specialized surface structures and unique material compositions, allowing biological species to passively regulate heat exchange while minimizing thermal stress. Despite the growing interest in bio‐inspired thermal management strategies, many biological species with radiative cooling mechanisms have not been sufficiently studied. Learning from these naturally evolved systems offers a distinct opportunity to develop novel radiative cooling technologies that surpass conventional engineering approaches. Notably, the intricate microstructures observed on the surfaces of certain insects exhibit exceptional thermal regulation capabilities. For example, the white beetle *Goliathus goliatus* exhibits a delicate shell with hollow cylindrical microstructure on its exocuticle, which enhances visible reflectivity and IR emissivity (Figure 3I).^[^ ^51^ ^]^ Specifically, the thin film structure on the exocuticle shows high visible reflectivity owing to thin‐film interference, while the internal air layers further enhance overall reflectivity through multi‐scattering effects. Furthermore, the chitin‐based structural absorption and anti‐reflective properties increase IR emissivity, which plays a vital role in thermal management by dissipating excessive heat.

Another notable example is a hierarchical microstructure on the hindwing scent patch scale of the *Rapala dioetas* butterfly, which shows enhanced IR emissivity for thermal management (Figure 3J).^[^ ^52^ ^]^ This enhancement results from the combined effects of the scale microstructures and their internal components. The hierarchical microstructures significantly increase the internal surface area, resulting in enhanced IR emissivity. Simultaneously, the presence of internal microfibers leads to multi‐scattering effects, increasing photon free path and the probability of IR emission. This structural adaptation enables *Rapala dioetas* to dissipate excess heat effectively from the scent patch region, ensuring a stable temperature for the underlying living cells involved in pheromone production.

These biological structures provide an effective model for the development of next‐generation materials with enhanced radiative cooling performance. Organisms that regulate heat in nature can effectively perform radiative cooling without relying on external energy sources, and leveraging these principles can significantly reduce the energy consumption of cooling systems. This approach offers significant potential for enhancing energy efficiency across buildings, electronics, and automotive applications. Furthermore, bio‐inspired designs leverage complex hierarchical structures, offering superior thermal radiation performance and significant opportunities for advanced thermal management solutions.

### 2.2 Thermal Regulation

To adapt to environments where temperatures are ever‐changing (i.e., diurnal temperature fluctuations or climates with seasonal variations), organisms have evolved dynamic and self‐adaptive behaviors. In this section, we introduce the intelligent thermal regulation strategies of biological organisms and review recent efforts to develop bio‐inspired thermal regulation systems.

#### 2.2.1 Visible and IR thermochromism inspired by Himalayan rabbit and mimosa

**Figure 4:**
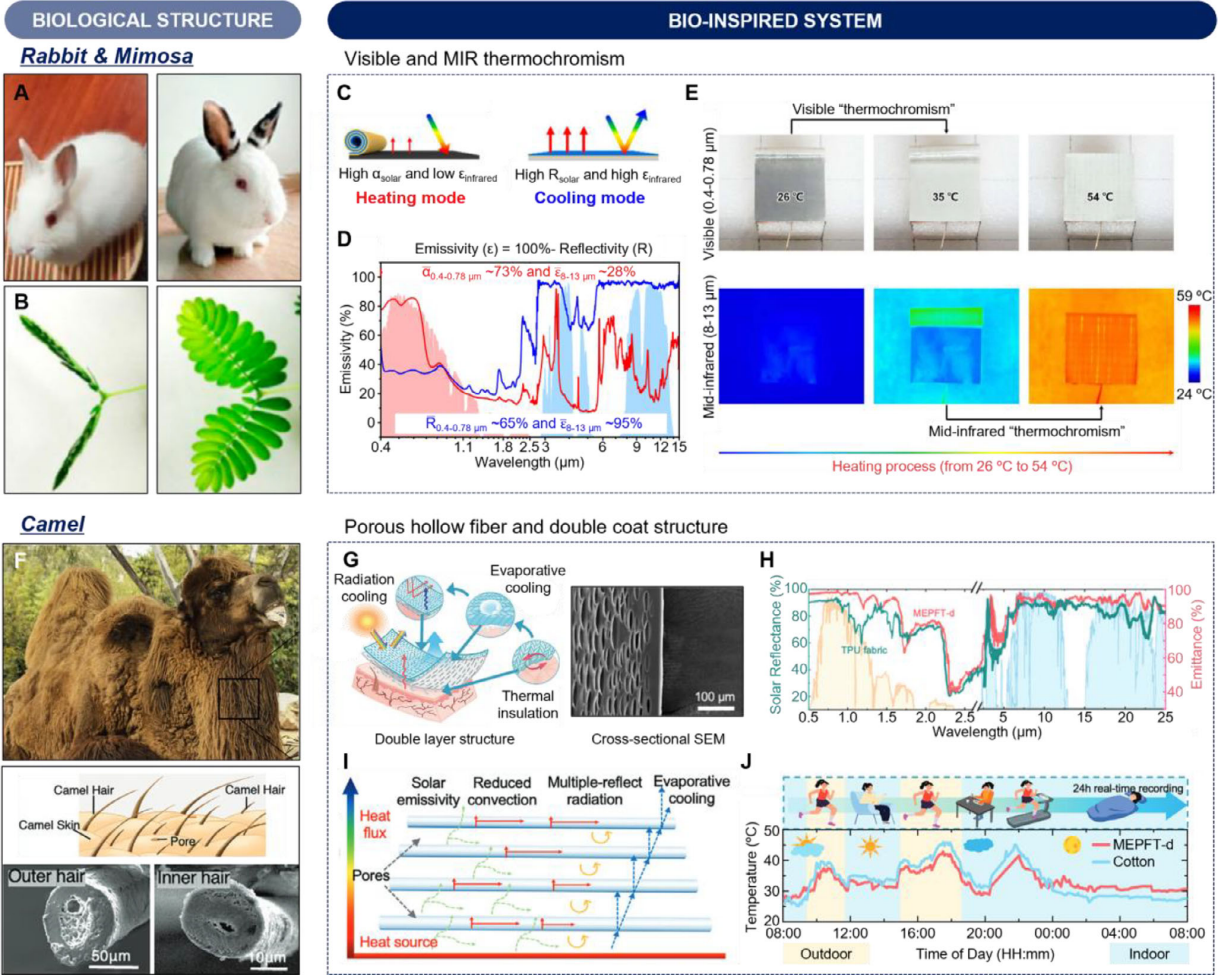
Biological and bio‐inspired structures for thermal regulation. A) Photograph of a Himalayan rabbit (*Oryctolagus cuniculus*), showing visible thermochromism of Himalayan rabbit hairs. B) Optical image of mimosa leaves (*Mimosa pudica*), presenting covering area variation. C) Schematic illustrations of spectral characteristics of dual‐mode thermal management device combining a thermochromic bottom layer with low IR emissivity and two‐way shape memory polymer top actuating layer with high IR emissivity. D) Optical properties of dual‐mode thermal management device in heating and cooling mode. E) Photograph and thermal images of dual‐mode thermal management device under heating conditions, highlighting visible and IR thermochromism. Reproduced with permission.^[^ ^53^ ^]^ Copyright 2024 PNAS. F) Photograph of a camel (*Camelus* sp.) (top), schematic illustration of double hair coat structure (middle), and porous hollow fiber and double coat structure for radiative cooling and thermal insulation (bottom). Reproduced with permission.^[^ ^56^ ^]^ Copyright 2020, Elsevier. Reproduced with permission.^[^ ^57^ ^]^ Copyright 2024, Wiley‐VCH. G) Illustration of bioinspired porous fabric (left) and cross‐sectional SEM image of porous elastic TPU fiber (right). H) Solar reflectance and IR emittance of commercial TPU fabric and MEPFT‐d. I) Schematic diagram of heat transfer mechanism of MEPFT‐d. J) Real‐time temperature monitoring of cotton fabric and MEPFT‐d in all weather conditions. Reproduced with permission.^[^ ^57^ ^]^ Copyright 2024, Wiley‐VCH.

Biological organisms with their remarkable ability to adapt to ever‐changing environmental conditions provide innovative sources for bio‐inspired thermal‐management strategies. For example, the Himalayan rabbit (*Oryctolagus cuniculus*) shows the color change of hair by temperature (**Figure** **4**A).^[^ ^53^ ^]^ The color of the hair remains white when the weather is warm, whereas the hair of the Himalayan rabbit darkens in cold weather. These color changes in the hair of the Himalayan rabbit help maintain their body temperature within a comfortable range by adjusting solar‐thermal conversion efficiency. Another example is mimosa (*Mimosa pudica*) leaves, which show folding behavior induced by environmental stimuli such as warming (Figure 4B). The folding‐opening behavior of mimosa leaves not only reduces leaf damage but also enables functional regulation by modulating the photosynthesis rate.

Zhang et al. reported a dual‐mode thermal management device integrated into the thermal properties of the Himalayan rabbit hairs and mechanical properties of mimosa leaves, showing visible and IR thermochromism.^[^ ^53^ ^]^ As shown in Figure 4C, the device consists of a thermochromic bottom layer with low IR emissivity and a two‐way shape memory polymer top actuating layer with high IR emissivity. This distinctive structure modulates spectral characteristics in visible and IR wavelength regions, enabling thermal radiation control. In the cooling mode, the actuating layer exhibits an unfolding state, showing high solar reflectivity and high IR emissivity (Figure 4C; right). In contrast, the upper layer structure displays a folding state in the heating mode, resulting in high solar absorptivity and low IR emissivity (Figure 4C; left). The device in cooling mode achieves exceptional optical characteristics with an average reflectivity of 65% in the visible range and emissivity of 95% in the LWIR wavelength region (Figure 4D). Furthermore, in the heating mode, the device shows a 38% increase in solar absorptivity and a 67% decrease in IR emissivity. Figure 4E shows the optical (top) and thermal images (bottom) of the dual‐mode thermal management device by temperature. When the temperature of the heating plate increased from 26 to 35 °C, the thermochromic layer of the device exhibited visible thermochromism. Additionally, the device also shows IR thermochromism when the temperature of the heating plate attained 55 °C, which indicates that the dual‐mode thermal management devices could realize simultaneous temperature control without external energy consumption.

Extensive research efforts have been dedicated to the development of thermal management using thermochromic material structures. Zhang et al. developed a dual‐mode thermal management, consisting of solar heating layer, temperature‐sensitive actuating layer, and radiative cooling layer.^[^ ^54^ ^]^ This device exhibits solar reflectivity of 90% and IR emissivity of 97% for cooling mode, and solar absorptivity of 91% and IR emissivity of 8% for heating mode, achieving an average heating temperature of 6 °C and cooling temperature of 15 °C compared to ambient air. Furthermore, a thermochromic hydrogel integrated with a solar absorber has been reported for dynamic thermal regulation.^[^ ^55^ ^]^ The adaptive reflectance‐switchable hydrogel emitter exhibited dynamic solar reflectivity variation of 75% between heating and cooling modes and static IR emissivity of 95%, demonstrating excellent thermal regulation performance at the target temperature. These thermochromic properties highlight the feasibility of passive temperature control across various environmental conditions and a promising material system for autonomous seasonal thermal regulation.

#### 2.2.2 Porous hollow fiber and double coat structure inspired by camel

Some mammals have evolved specialized coat structures to maintain their body temperature against significant diurnal temperature variations in the desert. A representative example is the camel (*Camelus* sp.), which has developed a double coat structure with porous hollow hairs to adapt to the high solar radiation and substantial temperature fluctuations characteristic of desert climate (Figure 4F).^[^ ^56^, ^57^ ^]^ The outer layer features thick and porous hair, providing superior vis–NIR reflectivity and IR emissivity. In contrast, the inner layer of thin and porous hair reduces the heat conductive path, thereby enhancing the heat‐insulating effect induced by the limitation of heat conduction and convection. Furthermore, this porous structure facilitates breathability, contributing to evaporative cooling and thus maximizing cooling efficiency. This unique double coat structure with porous hollow hairs of camels exhibits a synergistic effect by radiative and evaporative cooling during the daytime, while it demonstrates heat insulation properties at nighttime, enabling effective thermal management.

**Figure 5:**
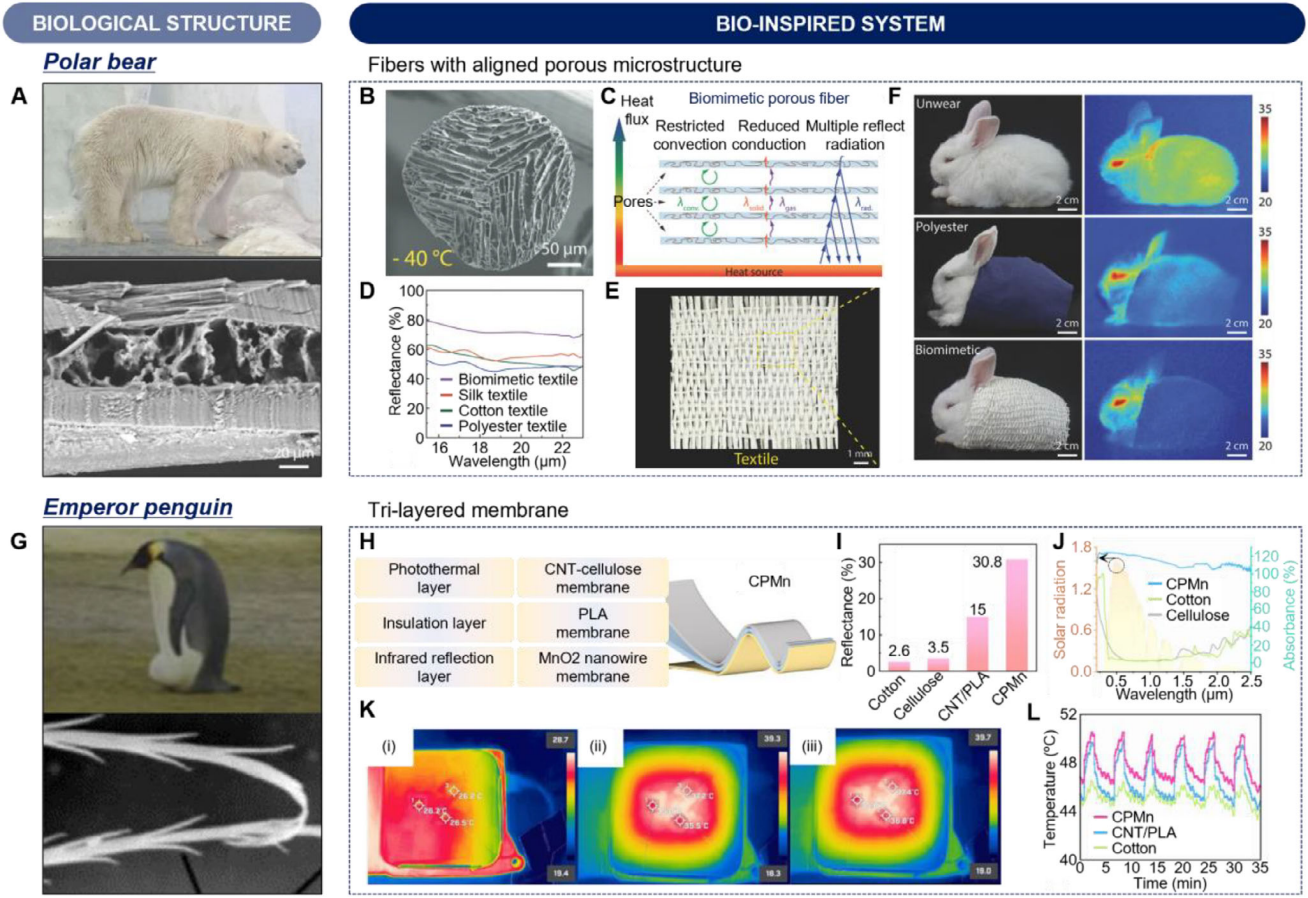
Biological and bio‐inspired porous structures for thermal insulation. A) Photograph of a polar bear (*Ursus maritimus*) (top) and cross‐sectional SEM image of a single fur on a polar bear, featuring aligned porous microstructure (bottom). B) Cross‐sectional SEM image of aligned porous structure of single fiber using silk fibroin/chitosan mixed solution by freeze‐spinning technique, combining solution spinning and directional freezing. C) Schematic illustration of heat transfer mechanism and thermal conductivity of the fabricated porous fiber with aligned pores. *λ*_conv._, thermal convection; *λ*_solid_, solid conduction; *λ*_gas_, gas conduction. *λ*_rad._, thermal radiation. D) Optical properties of commercial silk, cotton, polyester, and bio‐inspired textiles. E) Optical image of a bio‐inspired woven porous fiber, showing the potential for large‐scale fabrication. F) Optical and thermal images of rabbits wearing different materials: unworn, commercial polyester, and biomimetic textiles. The biomimetic textile shows thermal stealth property. Reproduced with permission.^[^ ^30^ ^]^ Copyright 2018, Wiley‐VCH. G) Photograph of an emperor penguin (*Aptenodytes forsteri*) (top) and SEM image (bottom) of microstructure of down feathers adapted to extreme cold environments, leading to thermal insulation. The Royal Society. Reproduced with permission.^[^ ^14^ ^]^ Copyright 1999, Academic Press. Reproduced with permission.^[^ ^15^ ^]^ Copyright 2013, Royal Society. H) Schematic illustrations of theCNT/PLA/MnO_2_ (CPMn) membrane. The CPMn membrane consists of three layers of photothermal layer (CNT‐cellulose membrane), insulation layer (PLA membrane), and IR reflection layer (MnO_2_ nanowire membrane). I) IR reflectance of cotton, cellulose membrane, CNT/PLA membrane, and CPMn. J) Solar radiation and absorbance spectra of cotton, cellulose membrane, and CPMn membrane in the solar wavelength range. K) Thermal images of (i) CPMn membrane, (ii) CNT/PLA membrane, and (iii) cotton after stabilizing temperature. L) Repeated thermal cyclic test of CPMn (pink line), CNT/PLA membrane (blue line), and cotton (green line). Reproduced with permission.^[^ ^61^ ^]^ Copyright 2024, Springer Nature.

Inspired by the hair structure of camel for thermal regulation, Wang et al. developed a double‐layered fabric using micro‐extruded physically foamed porous elastic fiber with TPU elastomer (MEPFT‐d), showing elongated micro‐nano‐pore structure (Figure 4G).^[^ ^57^ ^]^ As shown in Figure 4G, the thermal regulation mechanisms of MEPFT‐d incorporate three primary strategies. The micro‐ and nano‐sized pores enhance solar reflectivity and IR emissivity, enabling radiative cooling during the daytime. Furthermore, the double‐sided and elongated micro–nano‐pore structure provides unidirectional water transport channels, which accelerate evaporative cooling and dissipation of the heat from the skin. At nighttime, the thermal insulation mechanism is facilitated by the porous structure of the fabric, which significantly lowers thermal conductivity and acts as an insulator, reducing heat input. Figure 4H shows the solar reflectivity and IR emissivity of TPU fabric and MEPFT‐d bilayer fabric. The MEPFT‐d bilayer fabric exhibits solar reflectivity of 98.7%, which is higher than that of TPU fabric (83.2%). Furthermore, the MEPFT‐d bilayer shows an IR emissivity of 97.2%, enabling superior radiative cooling performance. Based on these superior structural and optical characteristics, the heat transfer mechanism of MEPFT‐d can be summarized in Figure 4H. The MEPFT‐d has a high solar reflectivity by the interference effect of micro–nano‐pore structure and IR emissivity, enabling excellent radiative cooling performance. Furthermore, the gradient micro–nano‐pore structure of MEPFT‐d provides unidirectional wet conductivity, which dissipates heat from the skin by evaporative cooling. Moreover, the MEPFT‐d also exhibits low thermal conductivity, which facilitates thermal insulation against ambient temperature. As shown in Figure 5J, the MEPFT‐d demonstrated more stable thermal regulation effects compared to cotton, depending on external environmental changes. The synergistic cooling effects combined with thermal radiation and evaporation, as well as the thermal insulation properties of the MEPFT‐d, can be used for personal thermal management applications.

### 2.3 Thermal Insulation

Biological organisms living in cold environments, such as the Arctic or Antarctic, have evolved various biological systems to reduce heat loss. Among these systems, trapping air serves as thermal insulation properties, enabling the simultaneous suppression of heat transfer through conduction, convection, and radiation. In this section, we introduce the detailed biological air‐trapping structures and thermal insulation properties of animals and also explore recent advancements in bio‐inspired thermal insulation systems that mimic these structures.

#### 2.3.1 Aligned porous fiber aerogel inspired by poplar bear

In nature, for mammals inhabiting extremely cold environments, such as polar bears (*Ursus maritimus*), minimizing heat loss is crucial. The polar bear exhibits thermal insulation properties through its thick fat layer and unique hair structure featuring a hollow core and aligned shell, as depicted in **Figure** **5**A.^[^ ^16^, ^30^ ^]^ Specifically, the polar bear utilizes its hollow hair structure to reflect IR radiation emitting from the skin, and the trapped air within the hollow hair structure reduces heat convection and conduction, thereby minimizing heat loss. Consequently, fibers designed to mimic the microstructure of polar bear hairs are the focus of attention as thermal management applications.

Inspired by the microstructure of polar bear hairs, Ying et al. fabricated a textile woven with bio‐inspired aerogel fibers as thermal insulating material using the freeze‐spinning method (Figure 5B).^[^ ^30^ ^]^ The bio‐inspired fibers exhibit a tailored porous structure with a high porosity of 87%. Figure 5C presents the heat transfer mechanism of the bio‐inspired textile. Theoretically, the thermal conductivity of the bio‐inspired textile is calculated by the sum of the thermal convection (λ_conv_), solid thermal conduction (λ_solid_), air thermal conduction (λ_gas_), and thermal radiation (λ_rad_). In bio‐inspired textiles, the λ_conv_ is significantly blocked by the porosity within the textile, and the presence of air within the pores results in low thermal conductivity (λ_solid_ > λ_gas_), which leads to superior thermal insulating properties. Furthermore, the bio‐inspired textile can significantly reflect the IR wavelength region by the multiple solid–air interfaces of the textile. As shown in Figure 5D, the bio‐inspired textile exhibits the highest average IR reflectance of 70–80% compared to other commercial textiles. Moreover, as shown in Figure 5E, the bio‐inspired textile demonstrates the potential for scalable fabrication using a freeze‐spinning technique suitable for human thermal management applications. Based on these optical properties, bio‐inspired textiles not only provide thermal insulation properties but also offer thermal stealth characteristics. Furthermore, the bio‐inspired textile is almost invisible under an IR camera and exhibits a surface temperature similar to the ambient air temperature compared to polyester (Figure 5F).

Achieving thermal insulation by mimicking the structure of polar bear fur is essential, but it's also crucial to consider the mechanical characteristics that play a key role in human thermal applications such as clothing. Additionally, the superior thermal insulation properties demonstrated can potentially be utilized for protective clothing in high‐temperature environments, highlighting the multifaceted utility of these innovations. Wang et al. reported a textile woven with polyimide (PI) aerogel fibers.^[^ ^58^ ^]^ The fabricated PI aerogel fibers exhibited an excellent tensile strength of 14.7 MPa due to the aligned porous structure within fibers. Additionally, while polyester reached its melting point (250 °C) at an ambient temperature of 300 °C, the bio‐inspired textile maintained a lower temperature of 210.5 °C, demonstrating its thermal insulating performance even in high‐temperature environments. Moreover, a stretchable TPU fiber with ultrahigh stretchability (1468%) and thermal insulation was designed.^[^ ^59^ ^]^ In extreme environments of −40 °C and 115 °C, it achieved absolute temperature differences of 68 °C and 44 °C, respectively, confirming its adaptability in both cold and hot conditions.

#### 2.3.2 Aerogel with aligned porous channel inspired by Emperor penguin

Emperor penguin (*Aptenodytes forsteri*) is another example that thrives in extremely harsh environments owing to the complex structure of its feathers (Figure 5G).^[^ ^14^, ^15^, ^60^ ^]^ The feathers of emperor penguin features a hierarchically branched structure attached to the main rachis. Furthermore, the branched structure is composed of long barbs, which consist of barbules.^[^ ^16^ ^]^ This unique hierarchical structure of feathers enables the penguin to trap air, resulting in low thermal conductivity. Additionally, the trapped air effectively reflects IR radiation emitted from the skin, thereby achieving high thermal insulation properties in extremely cold environments. Furthermore, the outermost parts of feathers are characterized by black tips, which absorb solar radiation to warm the air layer under the feathers.

Drawing inspiration from the natural thermally protective strategy of penguin feathers, Ran et al. designed carbon nanotube (CNT)/polylactic acid (PLA)/manganese dioxide (MnO_2_) (CPMn) multilayer membrane (Figure 5H).^[^ ^61^ ^]^ The external layer was fabricated by CNT‐cellulose solution to mimic the black tip of a penguin's feather, providing exceptional photothermal conversion. To trap a large amount of air for effective thermal insulating performance, the PLA fiber was loaded by electrospinning to mimic the middle part of the feather. Additionally, the interior layer, consisting of a MnO_2_‐cellulose membrane, effectively reflects IR radiation from the skin of the penguin to minimize heat loss. As shown in Figure 5I, the IR reflectivity of CPMn exhibits 30.8%, which marks a significant enhancement compared to 2.6% and 3.5% for cotton and cellulose membranes, respectively. Additionally, the IR reflectivity of the CNT/PLA membrane (i.e., CPMn without MnO_2_‐cellulose membrane) is only 15%, demonstrating that the incorporation of MnO_2_‐cellulose membrane substantially improves the IR reflectivity of the overall structure. Furthermore, the absorptivity of CPMn within the solar wavelength range is higher compared to commercial cotton and cellulose membranes, demonstrating the capability for a photothermal conversion effect similar to the black tip of penguin feather (Figure 5J). The thermal images provide additional evidence of thermal insulation properties by measuring the surface temperature of each membrane (CPMn, CNT‐cellulose/PLA membrane, and commercial cotton) on a hot plate set to 40 °C (Figure 5K). The surface temperature of CPMn is observed to be lower than that of the CNT‐cellulose/PLA membrane and cotton, confirming that IR radiation emitted by the body is reflected by the MnO_2_‐cellulose membrane layer rather than being dissipated to the environment. These thermal insulation properties of CPMn are also confirmed to remain constant without material degradation through repeated measurements (Figure 5L). Moreover, the slower rate of temperature change in response to external environmental fluctuations, as observed with CPMn compared to CNT‐cellulose/PLA membrane and cotton, minimizes the risk of significant temperature variations that could induce potential health concerns, making it more comfortable to wear for users.

## 3 Thermal Utilization in Biological Systems and Bio‐Inspired Engineering of Materials

### 3.1 Moisture Harvesting

Water scarcity imposes serious challenges for survival in arid or semi‐arid climates, where certain insects and plants have developed the ability to collect water through evolutionary processes under these extreme environments.^[^ ^62^ ^]^ Among the technologies that develop nature‐inspired systems by imitating such natural phenomena, the integration of IR radiation stands out as one of the innovative approaches. This section explores mechanisms of water harvesting in nature, and exampled applications through bio‐inspired technologies.

#### 3.1.1 Hydrophilic‐hydrophobic patterned surface inspired by Namib black beetles

**Figure 6:**
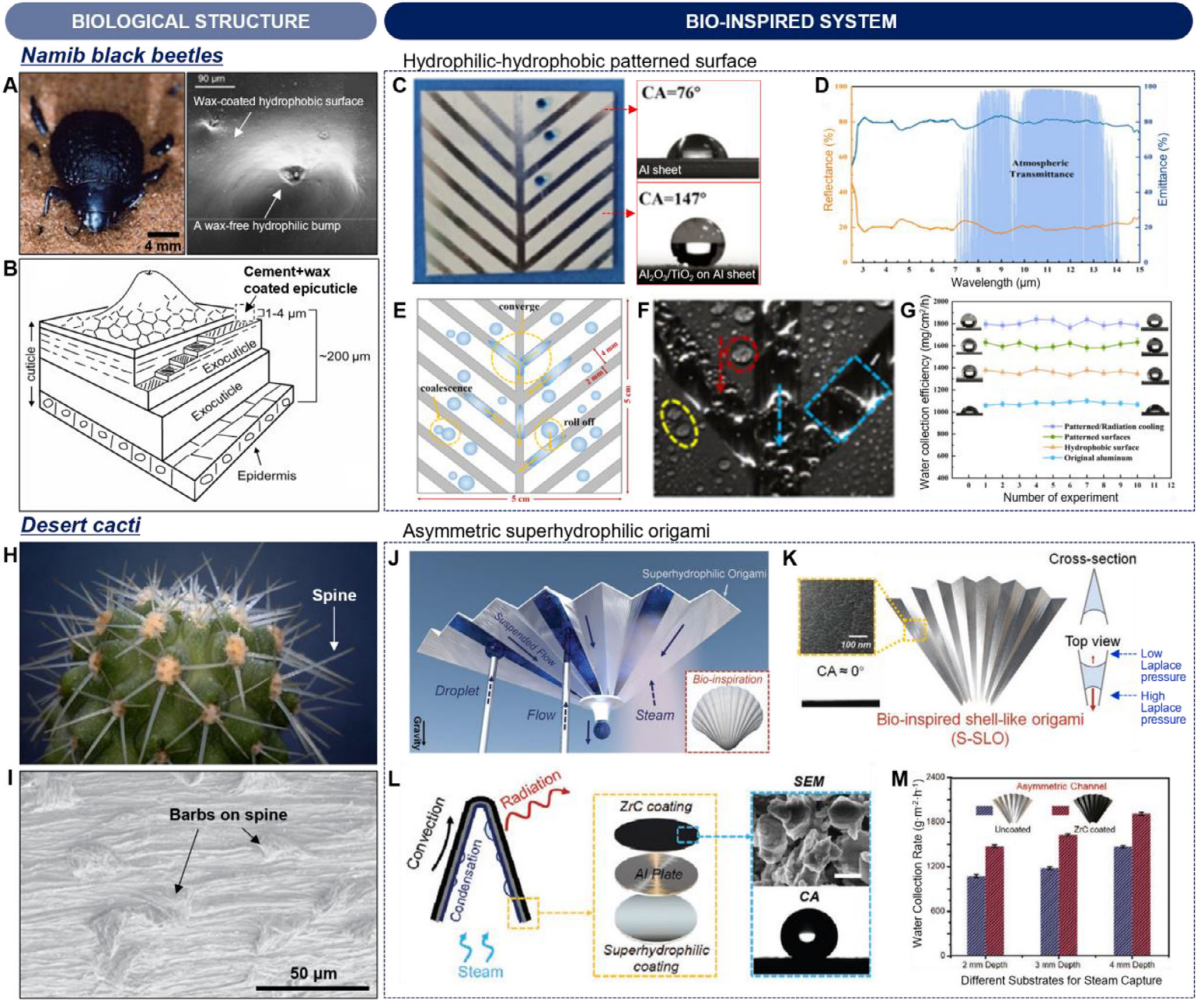
Biological and bio‐inspired structures for water harvesting. A) Optical image of a Namib black beetle (*Physasterna cribripes*) (left) and SEM image of the beetle's elytra, featuring a wax‐free hydrophilic bump and a surrounding wax‐coated hydrophobic/radiative cooling surface (right). B) Cross‐sectional view of the elytra structure. Reproduced with permission.^[^ ^64^ ^]^ Copyright 2014, Springer Nature. C) Optical image of a hydrophilic–hydrophobic patterned surface, created by coating Al_2_O_3_ and TiO_2_ powders on an Al sheet. D) High IR emissivity of the other side of the Al sheet, coated with MgHPO_4_·0.78H_2_O/ P(VDF‐HFP). E,F) Schematic (E) and photograph (F) of dew condensation and growth processes on the patterned surface. G) Comparative graph of water collection efficiencies for various surfaces. Reproduced with permission.^[^ ^65^ ^]^ Copyright 2023, Elsevier. H) Optical image of a desert cactus (*Copiapoa cinerea*) featuring hydrophilic/radiative cooling spines on stems. I) SEM image of the spine mid‐section. Reproduced with permission.^[^ ^68^ ^]^ Copyright 2015, IOP Publishing. J) A S‐SLO capable of spontaneous moisture capture and unidirectional water transport. K) Optical image of the S‐SLO made from an Al plate modified with a superhydrophilic reagent, where a Laplace pressure gradient along an asymmetric origami channel induces unidirectional water flow. L) Coating of a ZrC/PDMS composite on the outer surface of S‐SLO for radiative cooling. M) Vapor condensation performances of S‐SLOs with varying channel depths and radiative cooling effect. Reproduced with permission.^[^ ^69^ ^]^ Copyright 2023, Wiley‐VCH.

Namib Desert beetles have developed a highly efficient fog‐harvesting strategy to survive in one of the world's driest environments.^[^ ^33^, ^63^ ^]^ Their backs are covered with a specialized surface featuring a unique pattern of wax‐free hydrophilic bumps and wax‐coated hydrophobic valleys (**Figure** **6**A).^[^ ^64^ ^]^ By positioning themselves against the foggy winds, water droplets condense on the hydrophilic bumps and, once enough water accumulates, the droplets roll down the hydrophobic channels directly into the beetle's mouth. This process is further enhanced by high IR emissivity (95% ± 7% within ATW) of the wax layer, located on the top of the epicuticle, which promotes radiative cooling and facilitates dew condensation, improving water collection efficiency (Figure 6B).

Such an interesting moisture collection strategy has inspired numerous technological innovations for addressing global water scarcity challenges. A standout example involved the creation of a hydrophilic–hydrophobic patterned surface by coating Al_2_O_3_ and TiO_2_ powders on an aluminum (Al) sheet (Figure 6C).^[^ ^65^ ^]^ This vein‐like patterning imitated the Namib Desert beetle's method of collecting atmospheric moisture, employing zones that selectively attract and repel water to optimize condensation and collection efficiency. In addition to the surface patterning, the reverse side of the Al sheet was coated with magnesium phosphate hydrate (MgHPO_4_·0.78H_2_O) and P(VDF‐HFP), creating a high IR emissivity surface (Figure 6D). This radiative layer boosted the condensation process by effectively lowering the surface temperature. Figure 6E,F illustrate the water collection mechanism. Water droplets are nucleated and grew by condensation, merging with neighboring droplets on the hydrophobic regions. As droplets grew, gravity allowed them to roll toward and converge in the hydrophilic channels, guiding the water to a central collection point. A comparative analysis of water collection efficiencies in Figure 6G highlighted the superiority of the biomimetic surface over pristine Al sheet and those without hydrophobic or radiative cooling treatments. This biomimetic design markedly improved water collection efficiency to a value of 1803.12 mgcm^−2^h^−1^, with stable performance over multiple cycles.

Beyond the hydrophilic–hydrophobic patterned Al surface, several studies have explored alternative strategies to enhance condensation and collection efficiency. For instance, Haechler et al. developed a radiative cooling‐assisted system for continuous 24‐h water collection, combining passive superhydrophobic surfaces with coalescence‐induced water removal.^[^ ^66^ ^]^ Another study introduced a multifunctional coating of hydrophobic SiO₂ and hydrophilic TiO₂ nanospheres, leveraging wettability engineering and radiative cooling to boost dew condensation.^[^ ^67^ ^]^

#### 3.1.2 Asymmetric superhydrophilic origami inspired by desert cacti

Desert cacti, e.g., *Copiapoa cinerea*, utilize hydrophilic surfaces, radiative cooling, and specialized physical structures to optimize dew collection (Figure 6H).^[^ ^68^ ^]^ During the night, radiative cooling lowers the surface temperature below ambient levels, promoting the condensation of atmospheric moisture. The spines, which often feature hydrophilic surfaces, serve as primary sites for dew collection, efficiently capturing water vapor and facilitating droplet formation. A critical feature in this process is the presence of barbs along the spines, which generate a directional capillary effect that propels water droplets toward the stem (Figure 6I). This movement is further enhanced by a Laplace pressure gradient, which arises due to the conical shape and varying curvature of the spines. As water droplets accumulate at the spine tips, the pressure difference naturally guides them downward, ensuring efficient water transport to the plant's core for absorption and storage.

Inspired by the passive yet highly effective water collection mechanisms, Bai et al. developed a shell‐like superhydrophilic origami (S‐SLO) structure capable of spontaneous moisture capture and unidirectional water transport (Figure 6J).^[^ ^69^ ^]^ The device consisted of an Al plate modified with a superhydrophilic reagent, forming an asymmetric origami channel that enhanced water transport efficiency (Figure 6K). A key feature of this structure was the Laplace pressure gradient, generated by the asymmetric shape of the origami channels. This pressure differential actively propelled condensed water in a single direction, minimizing retention losses and improving collection efficiency. To further enhance condensation performance, the outer surface of the S‐SLO was coated with a zirconium carbide (ZrC)/PDMS composite layer (Figure 6L). This coating exhibited high IR emissivity (>80% in the 2.5–25 µm wavelengths), which facilitated passive heat dissipation and lowered the surface temperature, thereby increasing vapor condensation efficiency. Figure 6M shows a comparative analysis of water collection rate across different S‐SLO designs. The study revealed that channel depths and radiative cooling layer played crucial roles in water harvesting efficiency. Consequently, the optimized S‐SLO structure achieved up to a 56% increase in water collection performance compared to unmodified surfaces, highlighting the potential of combining surface chemistry, geometry‐driven unidirectional liquid transport, and passive thermal management for advanced water harvesting systems without the need for external energy input.

### 3.2 Camouflage

Camouflage is a crucial survival mechanism in biological systems, enabling organisms to blend into their environment to evade predators or ambush prey. Natural camouflage strategies include color adaptation, texture modulation, transparency, and dynamic pattern changes, observed in species such as cephalopods, chameleons, and certain insects.^[^ ^22^, ^70^, ^71^ ^]^ These organisms achieve camouflage through specialized skin structures, chromatophores, and light‐reflecting nanostructures, allowing for rapid and adaptive concealment. This section illustrates the intersection of camouflage in biological systems and its translation into innovative thermal camouflage technologies.

#### 3.2.1 Stretchable copolymer multilayer inspired by pelagic octopus and glass squid

**Figure 7:**
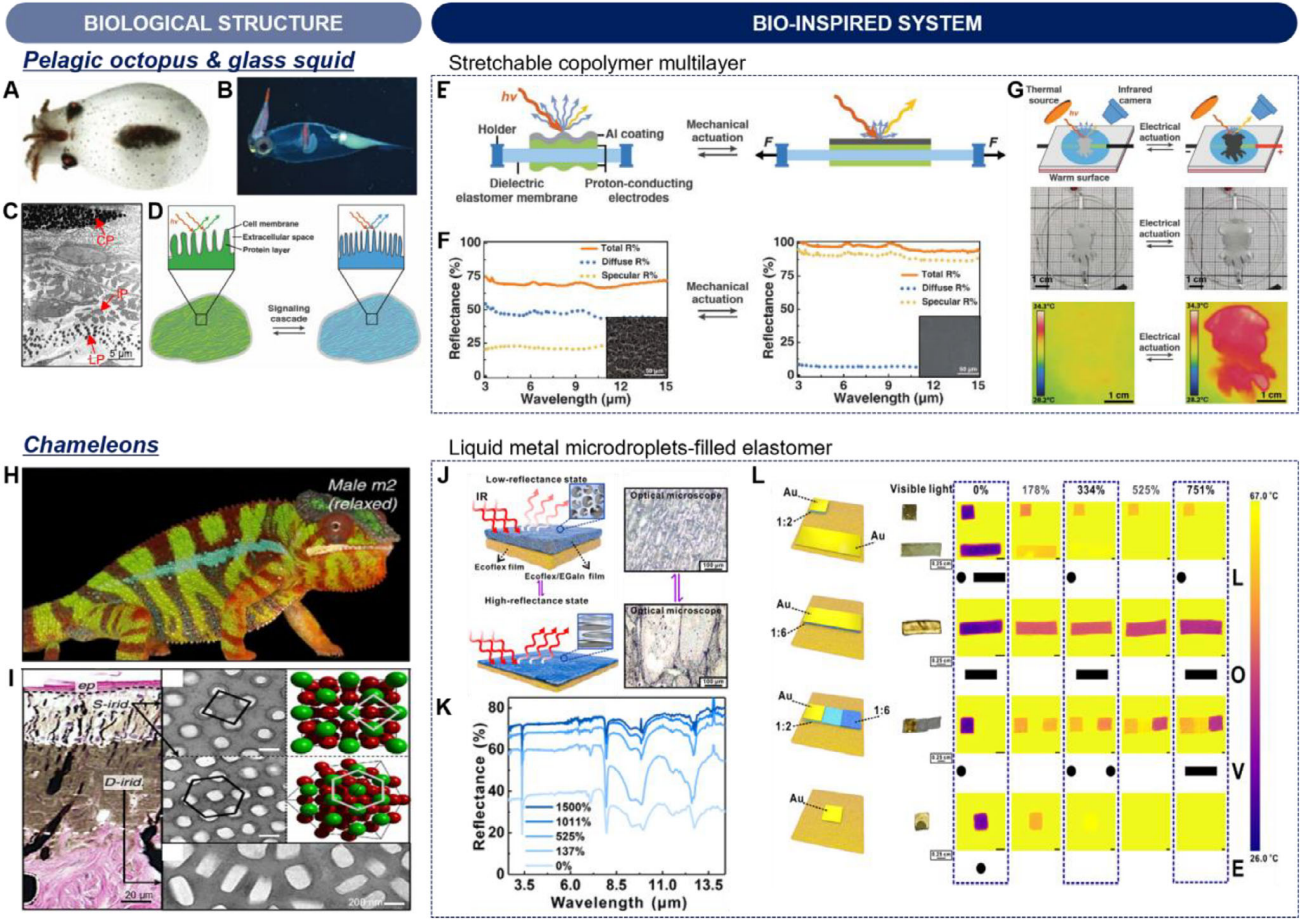
Biological structures for IR modulation and their applications. A,B) Photographs of representative pelagic octopuses (*Japetella healthi*) (A) and glass squids (*Taonius borealis*) (B) in their transparent state. Reproduced with permission.^[^ ^73^ ^]^ Copyright 2020, Wiley‐VCH. C) Cross‐sectional TEM image of pelagic octopus skin, with labels CP, IP, and LC denoting chromatophore, iridophore, and leucophore, respectively. Reproduced with permission.^[^ ^76^ ^]^ Copyright 2001, Wiley‐VCH. D) Schematic of the alternating arrangement of membrane‐enclosed protein layers and extracellular spaces in iridocytes, functioning as biological Bragg stacks for UV–vis and IR reflection. E,F) Illustration (E) and IR spectra with an SEM image (F) of a cephalopod‐inspired adaptive IR‐reflecting device, consisting of an acrylic dielectric elastomer membrane laminated with proton‐conducting electrodes on both top and bottom, and an Al film coating on the top electrode, before and after mechanical actuation. The actuation induced morphology changes and altered IR reflectance. G) Schematic (top), optical images (middle), and thermograms (bottom) of a squid‐shaped device before (left) and after (right) electric field‐induced mechanical actuation under constant thermal flux. Reproduced with permission.^[^ ^77^ ^]^ Copyright 2018, AAAS. H) Image of a panther chameleon (*Furcifer pardalis*). I) Cross‐sectional view of chameleon skin stained with haematoxylin and eosin (H&E) (right), and TEM images of guanine nanocrystals in superficial (S) and deep (D) iridophores, including a 3D FCC lattice model in the insets (left). Reproduced with permission.^[^ ^22^ ^]^ Copyright 2015, Springer Nature. J) Schematic and optical images of an IR‐modulating device featuring a bilayer of Ecoflex/EGaIn and Ecoflex before (top) and after (bottom) applying areal strain. The strain transformed the morphology of EGaIn droplets embedded in Ecoflex into a flake‐like shape, thereby enhancing IR reflectance. K) IR reflectance spectra of the device under various areal strains (0%, 137%, 525%, 1011%, and 1500%). L) Demonstration of IR Morse coding through applying predefined areal strains to specifically designed trilayers of Ecoflex/Ecoflex:EGaIn (1:2 and 1:6 in mass ratio)/Au. Reproduced with permission.^[^ ^78^ ^]^ Copyright 2024, Springer Nature.

Pelagic cephalopods, such as the pelagic octopus and glass squid, have evolved sophisticated camouflage strategies that allow them to remain nearly invisible in the open ocean (**Figure** **7**A,B).^[^ ^72^, ^73^ ^]^ Unlike benthic species that rely on background‐matching coloration,^[^ ^74^, ^75^ ^]^ these cephalopods utilize a combination of chromatophores (CPs), iridophores (IPs), and leucophores (LPs) to control light absorption, reflection, and scattering, enabling them to adapt to varying optical conditions (Figure 7C).^[^ ^76^ ^]^ At the core of their camouflage system, CPs contain expandable pigment sacs that adjust skin color in real time. Beneath these, IPs use alternating layers of membrane‐enclosed proteins to create structural coloration through thin‐film interference, selectively reflecting visible and near‐infrared light (Figure 7D).^[^ ^77^ ^]^ Complementing these layers, LPs function as broadband scatterers, reflecting ambient light across all wavelengths to enhance transparency and disguise. By fine‐tuning these optical elements, pelagic cephalopods achieve adaptive infrared camouflage, minimizing their thermal signature to blend seamlessly into their surroundings.

Inspired by cephalopods, Xu et al. developed an adaptive IR‐reflecting device capable of dynamically modulating IR reflectance through mechanically induced morphological changes (Figure 7E).^[^ ^77^ ^]^ This biomimetic system comprised an acrylic dielectric elastomer membrane sandwiched between proton‐conducting electrodes on both the top and bottom, with an Al film coating on the top electrode. When actuated by an electric field, the mechanical deformation of the membrane altered its surface morphology, directly impacting IR reflectance properties. Figure 7F displays a shift in total IR reflectivity from ≈71% to ≈96%, accompanied by a transition in surface morphology from a wrinkled to a flattened state upon mechanical actuation, highlighting the efficient tunability of the system. To further demonstrate the biomimetic IR camouflage capability, a squid‐shaped adaptive IR device was fabricated and tested under constant thermal flux (Figure 7G). Before actuation, the device's thermal signature closely matched its background, making it nearly indistinguishable under IR visualization. However, the post‐actuation thermographic image showed a clear change in thermal emissivity, demonstrating the potential of this adaptive IR‐reflecting system for stealth technologies, thermal management solutions, and next‐generation dynamic infrared camouflage.

#### 3.2.2 Liquid metal microdroplets‐filled elastomer inspired by chameleons

Chameleons are renowned for their dynamic color‐changing ability, which is primarily driven by specialized iridophore cells in their skin (Figure 7H).^[^ ^22^ ^]^ These iridophores play a crucial role not only in visible light reflection but also in IR modulation, allowing chameleons to achieve both camouflage and thermoregulation. Chameleons possess two distinct layers of iridophores: superficial iridophores (S‐iridophores) and deep iridophores (D‐iridophores), each contributing differently to light and heat management (Figure 7I). S‐iridophores, located closer to the surface, contain a network of nanocrystalline guanine structures that can actively modulate the distance between crystals. This structural rearrangement enables chameleons to tune vis–NIR reflectivity, shifting their color across a broad spectrum. Below this layer, D‐iridophores consist of larger, more densely packed nanocrystals that reflect NIR and MIR radiation, acting as a passive thermal barrier. This dual‐layered system provides efficient IR modulation, helping chameleons control heat gain or loss while maintaining adaptive camouflage.

The dynamic IR modulation of chameleons has translated into many innovative technologies. Figure 7J shows a bioinspired system mimicking the functionality of iridophores in chameleons, where mechanical deformation altered IR reflectance properties by redistributing embedded materials.^[^ ^78^ ^]^ This IR‐modulating device featured a bilayer structure composed of Ecoflex elastomer and liquid metal, eutectic gallium‐indium (EGaIn), microdroplets. Before actuation, EGaIn droplets were dispersed in the Ecoflex matrix, maintaining a low‐reflectance state. However, upon mechanical stretching, the droplets underwent morphological transformation into a flake‐like structure, significantly enhancing IR reflectivity. Figure 7K illustrates the IR reflectivity spectra of the device under varying areal strains (0%, 137%, 525%, 1011%, and 1500%), demonstrating its capacity to finely regulate IR signatures through controlled strain application. A key innovation of this chameleon‐inspired technology was its potential for programmable IR communication, as demonstrated in Figure 7L. By applying predefined areal strains to a tri‐layered system (Ecoflex/Ecoflex:EGaIn in 1:2 and 1:6 mass ratios/Au), the system selectively altered its IR reflectance, allowing for encoding and decoding information in the IR spectrum. This approach could create numerous, distinct infrared patterns, which can be applicable to information encryption, thermal camouflage, and advanced sensing applications.

### 3.3 IR Sensation

IR sensation is a remarkable biological adaptation that allows certain organisms to detect thermal radiation, providing survival advantages in hunting, navigation, and environmental awareness.^[^ ^25^, ^79^ ^]^ Unlike visible light perception, IR sensation relies on specialized thermoreceptors that respond to temperature variations, enabling species such as pit vipers, vampire bats, and certain beetles to perceive heat signatures in their surroundings. This section explores such biological mechanisms and their translation into IR detection technologies.

#### 3.3.1 Biohybrid infrared sensor inspired by vampire bat & pit vipers

**Figure 8:**
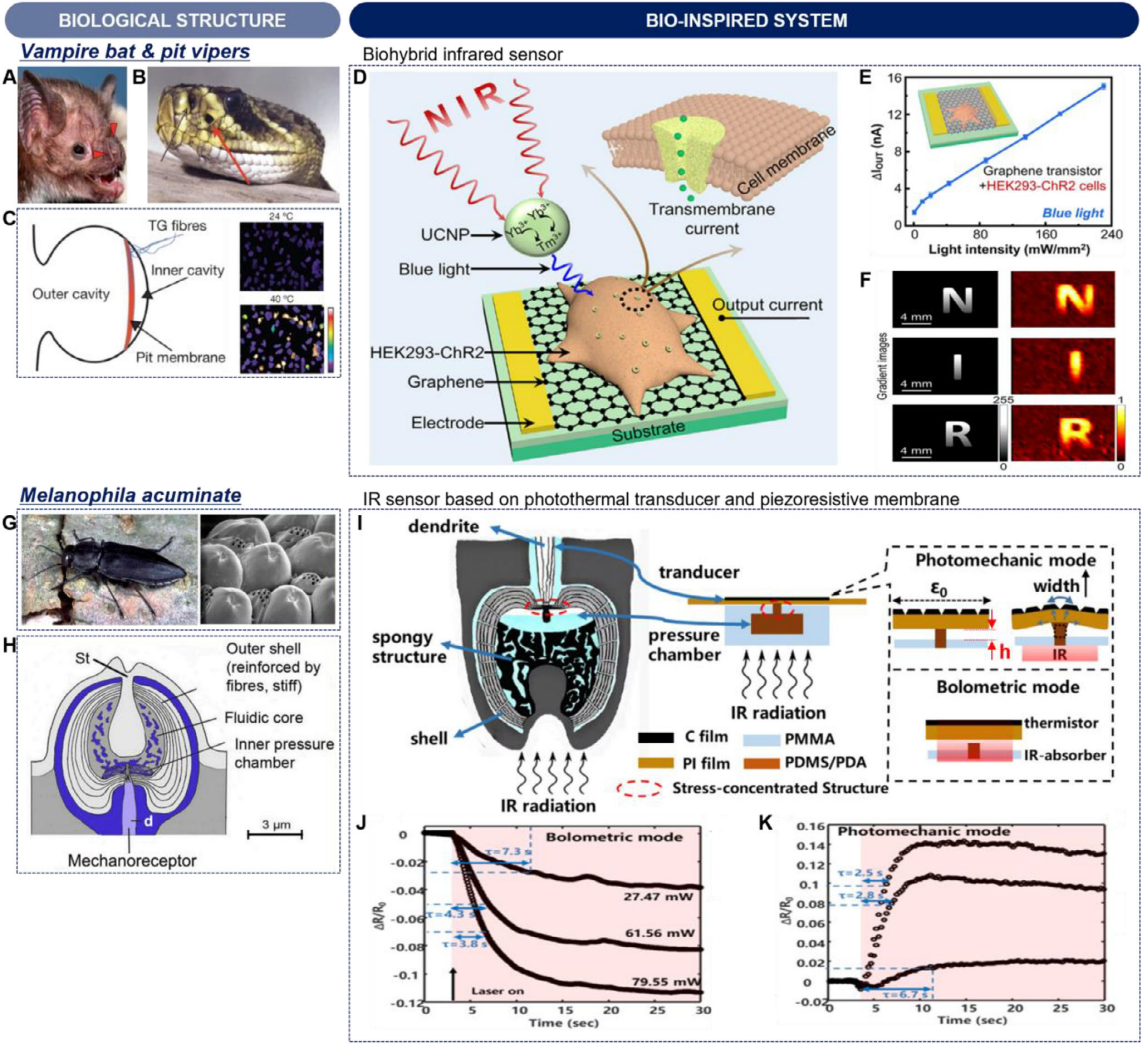
Biological and bio‐inspired structures for IR sensation. A) Facial anatomy of a vampire bat (*Desmodus rotundus*) with pit organs indicated by red arrowheads. Reproduced with permission.^[^ ^80^ ^]^ Copyright 2011, Springer Nature. B) Photograph of a rattlesnake (*Crotalus atrox*) with a loreal pit organ marked by a red arrow. C) Description of the structure of a pit organ (left), illustrating a pit membrane suspended within a cavity and connected to TG fibers, alongside calcium imaging (right) of heat‐evoked responses of rattlesnake TRPA1 channels) expressed in HEK293 cells. Reproduced with permission.^[^ ^81^ ^]^ Copyright 2010, Springer Nature. D) Schematic of a TRP‐inspired biohybrid IR sensor, composed of UCNPs that convert IR to blue light, HEK293 cells expressing ChR2 that generate a blue light‐responsive ion current, and a graphene transistor for recording cellular bioelectricity with ultrahigh carrier mobility and good biocompatibility. E) Demonstration of the photoelectric activity of HEK293‐ChR2 cells upon blue light irradiation. F) Examples of IR letters with a gray gradient (left) and their detection images by the biohybrid IR sensor (right). Reproduced with permission.^[^ ^82^ ^]^ Copyright 2023, Elsevier. G) Images of a “fire‐loving” beetle (*Melanophila acuminate*) (left) and a dome‐shaped sensilla array with small pores (right). Reproduced with permission.^[^ ^83^ ^]^ Copyright 2009, SPIE. H) Cross‐sectional description of an IR sensillum. Reproduced with permission.^[^ ^84^ ^]^ Copyright 2015, Frontiers. I) Illustration of a “fire‐beetle” pit organ‐inspired IR sensor, consisting of a polyimide (PI)/carbon piezoresistive sensing membrane with extremely high sensitivity and a PDA photothermal transducer, featuring a dendrite‐like tip protruding from a PMMA case. Adjusting the distance (h) between the sensing membrane and the dendrite tip enabled the sensor to operate in either photomechanic or bolometric modes. J,K) IR power‐dependent responses of the sensor in bolometric (J, h = 0 mm) and photomechanic (K, h = 1 mm) modes. Reproduced with permission.^[^ ^85^ ^]^ Copyright 2023, Elsevier.

Both vampire bats (*Desmodus rotundus*) and pit vipers (e.g., rattlesnakes, pythons) have evolved pit organs—a hollow or depression in an organ or the body—that act as biological IR sensors capable of monitoring temperature differences in the environment (**Figure** **8**A,B).^[^ ^80^, ^81^ ^]^ Pit vipers possess paired pit organs located between the eyes and nostrils, which contain a thin, highly vascularized membrane filled with heat‐sensitive nerve endings (Figure 8C).^[^ ^81^ ^]^ These nerve endings are densely packed with transient receptor potential (TRP) ion channels that respond to IR radiation in the 5–30 µm range, allowing snakes to form a thermal map of their surroundings. The pit organ structure serves as a natural pinhole camera capable of precise directional IR sensing, which improves prey detection and navigation even in total darkness. Similarly, vampire bats detect warm‐blooded prey with the ability to sense temperature differences using IR‐sensitive pit organs around their noses.

As a representative biomimetic technology, Figure 8D presents a biohybrid IR sensor consisting of upconversion nanoparticles (UCNPs), optogenetically engineered cells, and graphene transistors.^[^ ^82^ ^]^ The UCNPs served as IR‐to‐visible light converters, transforming IR radiation into blue light. This blue light then activated HEK293 cells engineered to express channelrhodopsin‐2 (ChR2), which generated a blue‐light‐responsive ion current. Finally, the ion current was recorded by a graphene transistor, which offered ultrahigh carrier mobility, ensuring efficient detection of bioelectric signals. Figure 8E exhibits the photoelectric activity of HEK293‐ChR2 cells upon blue light irradiation, confirming the successful conversion of IR stimuli into bioelectric signals. Figure 8F demonstrates the detection capability of IR‐encoded symbols. A set of IR patterns representing letters (“N”, “I”, and “R”) with a gray gradient (left) were projected onto the sensor, and the system successfully translated IR intensity variations into distinguishable optical signals, highlighting its potential for high‐resolution thermal imaging with applications in environmental monitoring and night vision.

#### 3.3.2 IR sensor based on photothermal transducer and piezoresistive membrane

The fire‐loving beetle *Melanophila acuminata* exhibits a remarkable ability to detect IR radiation, enabling it to locate forest fires from great distances (Figure 8G).^[^ ^83^ ^]^ This adaptation is critical for its survival, as the beetle relies on freshly burned wood to lay its eggs. *M. acuminata* detects IR radiation through highly specialized pit organs located on its thorax. These organs function as biological microbolometers, capable of detecting thermal radiation in the wavelength range of 3–5 µm, corresponding to peak emissions from wildfires. Each pit organ is lined with sensory neurons connected to dome‐shaped cuticular structures, which expand upon IR absorption (Figure 8H).^[^ ^84^ ^]^ This expansion generates a mechanosensory response, triggering neural signals that allow the beetle to locate heat sources from several kilometers away. The system's high sensitivity and rapid response make it one of the most finely tuned IR detection mechanisms found in insects, inspiring the design of innovative engineering applications.

For example, Figure 8I depicts a fire‐beetle‐inspired IR sensor that consists of a PI/carbon bilayer membrane for piezoresistive sensing and a PDMS/polydopamine (PDA) composite for photothermal transducer, enclosed within a polymethylmethacrylate (PMMA) chamber.^[^ ^85^ ^]^ The transducer featured a dendrite‐like tip that concentrated thermal expansion stress, similar to the mechanosensitive structures found in the beetle's natural IR receptors. The sensor's functionality was tunable by adjusting the distance (h) between the sensing membrane and the dendrite tip, allowing it to operate in either bolometric or photomechanic modes. In the bolometric mode (h = 0 mm), the sensor detected IR radiation by measuring temperature‐induced resistance changes in the carbon‐based piezoresistive membrane. This mode converted absorbed infrared energy into a thermal signal, enabling highly sensitive IR detection. Alternatively, in the photomechanic mode (h = 1 mm), the dendrite tip expanded thermally upon IR absorption, mechanically deforming the sensing membrane. This deformation generated a piezoresistive response, providing an alternative IR detection pathway that was independent of thermal effects. Figure 8J,K provide time responses of the sensor in both the sensing modes under varying IR power irradiation (27.47 mW, 61.56 mW, and 79.55 mW). The sensor exhibited superior responsivity and detection limits with values of −1.4 W⁻¹ and 0.03 mW in bolometric mode and 2.2 W⁻¹ and 0.23 mW in photomechanic mode, with a rapid response time. These high sensitivity and efficiency were comparable to commercial IR detectors, indicating the potential of the bio‐inspired IR sensor in miniaturized, low‐cost, and uncooled sensing technologies.

## 4 Other Applications

**Figure 9:**
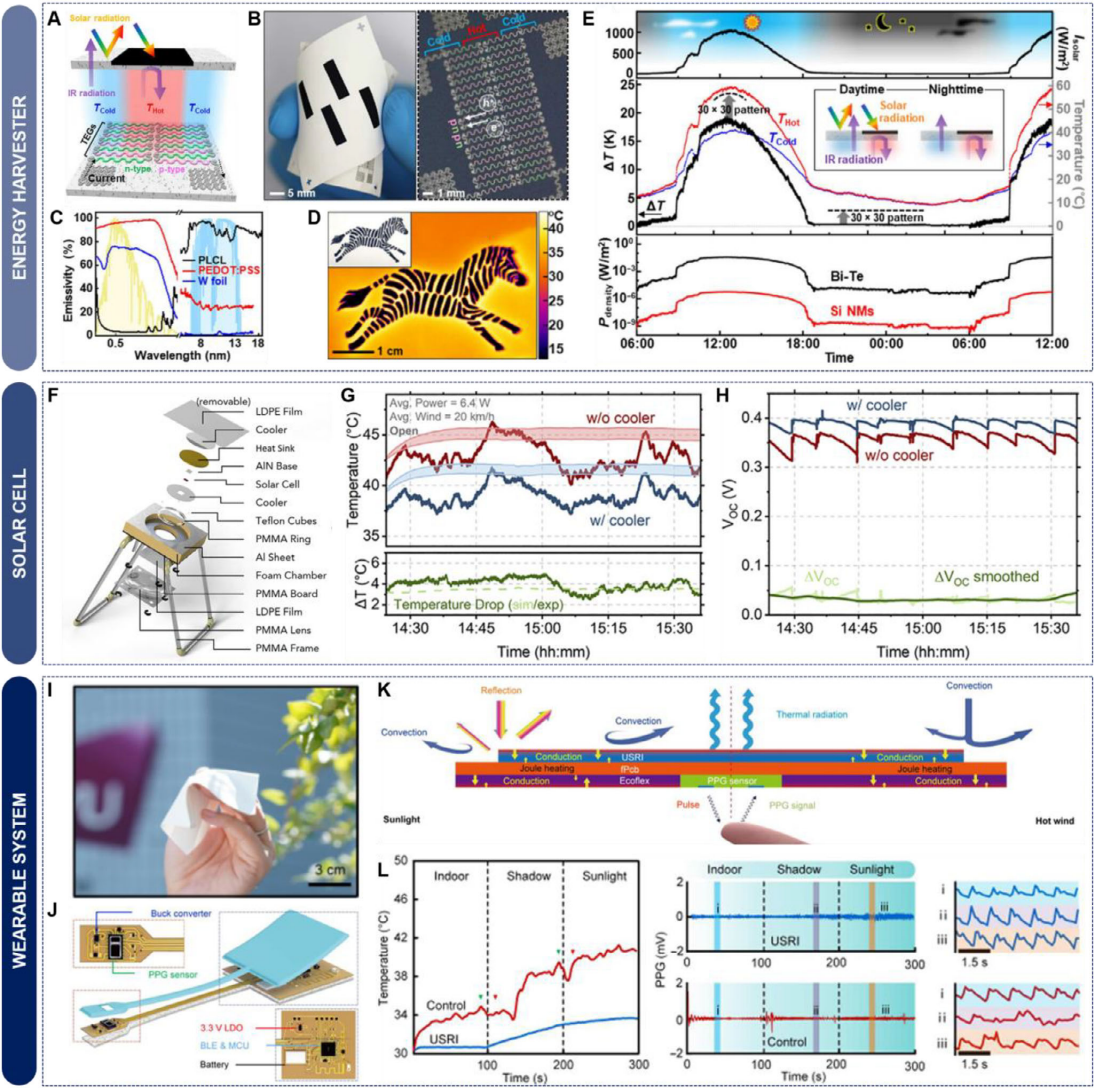
Management of IR radiation for electronic applications. A) Schematic of a stretchable, biodegradable energy harvester consisting of a top black/white patterned layer for generating an in‐plane thermal gradient throughout the day and a Si NMs‐based TEG layer for converting the thermal gradient into electricity. B) Optical images of the device showing the two layers (left) and arrays of n‐ and p‐type TEG legs (right). C) Emissivity spectra of PLCL, PEDOT:PSS, and W foil. (C) IR thermogram and corresponding optical image (inset) of a zebra stripes‐inspired radiative cooling/heating film, where the white PLCL microfibrous membrane exhibits high UV–vis reflectance and IR emissivity, while the dark patterns, made from conductive PEDOT:PSS film or W foil, provide high UV–vis absorption and IR reflectance. E) Real‐time measurements of solar irradiance, △T, and power density for the energy harvester with Si NMs‐ and Bi‐Te‐based TEGs. Reproduced with permission.^[^ ^86^ ^]^ Copyright 2023, AAAS. F) Exploded view of a CPV system integrated with two soda lime glass‐based radiative cooling layers. G,H) Measured (solid lines) and simulated (shaded areas and dashed line) temperatures of the CPV system with and without a radiative cooling layer (G), and simultaneous measurements of V_OC_, showing the radiative cooler enhanced voltage output by ≈28 mV. Reproduced with permission.^[^ ^87^ ^]^ Copyright 2020, Elsevie. I) Photograph of an USRI composed of hollow SiO_2_ microspheres, TiO_2_ nanoparticles, fluorescent pigments, and polystyrene‐acrylic. J,K) Schematics of the interface coated on a PPG sensing system (J) and its thermal exchange processes (K). L) Temporal measurements of temperatures (left) and PPG signals (middle and right) for the device with and without USRI under various environmental conditions (indoors, in shadow, and in sunlight). Reproduced with permission.^[^ ^88^ ^]^ Copyright 2023, AAAS.

In addition to the bioinspired technologies discussed in the previous sections, IR management offers a critical strategy for enhancing the performance of energy and electronic systems. As one such innovation, **Figure** **9**A illustrates a stretchable, biodegradable, energy harvesting system, which leveraged both thermal modulation (top layer) and thermoelectric generation (bottom layer).^[^ ^86^ ^]^ The zebra stripes‐inspired top layer was composed of the white poly(lactide‐ε‐caprolactone) (PLCL) microfibrous membrane, which exhibited high UV–vis reflectivity and strong IR emissivity, and the dark conductive poly(3,4‐ethylenedioxythiophene):poly(styrene sulfonate) (PEDOT:PSS) film or tungsten (W) foil, which provided high UV–vis absorptivity and IR reflectivity (Figure 9B). This combination generated an in‐plane thermal gradient, as confirmed by distinct thermographic profiles in Figure 9C. This thermal gradient drove charge separation in silicon nanomembrane (Si NM)‐based TEGs of the bottom layer, allowing direct heat‐to‐electricity conversion. Figure 9E presents real‐time energy scavenging performance over ≈1.5 days. The system generated continuous power outputs of <40 mW m^−2^ for bismuth telluride (Bi–Te)‐based TEGs and 0.006 mW m^−2^ for Si NMs‐based TEGs, demonstrating its potential as a scalable, sustainable energy solution.

Concentrating photovoltaics (CPV) operate under intensified solar radiation, making them highly susceptible to thermal degradation, which lowers power conversion efficiency and shortens device lifespan. As a promising approach to mitigate these problems, Figure 9F demonstrates integration of two soda lime glass‐based radiative cooling layers into a CPV system.^[^ ^87^ ^]^ These cooling layers took advantage of the ATW, allowing IR radiation to escape directly into space while reducing heat accumulation in the photovoltaic cells. To evaluate the cooling effectiveness, Figure 9G provides a comparison of measured and simulated temperature profiles for CPV systems with and without radiative cooling layers. The results indicated that the cooling layers significantly lowered operating temperatures, with reductions ranging from 5 °C to 36 °C depending on environmental conditions. This temperature reduction directly enhanced open‐circuit voltage (V_OC_) by at least 28 mV, corresponding to an 8% increase in voltage output (Figure 9H), which contributed to higher overall power generation efficiency while also prolonging the lifespan of the photovoltaic cells by mitigating thermal‐induced material degradation without relying on active cooling methods.

Thermal management strategy can also stabilize the performance of electronic devices. Figure 9I displays an ultrathin, soft, radiative cooling interface (USRI) capable of promoting radiative heat dissipation, maintaining mechanical flexibility, and achieving seamless integration for various skin‐interfaced electronic devices.^[^ ^88^ ^]^ The USRI was composed of hollow SiO₂ microspheres, TiO₂ nanoparticles, fluorescent pigments, and polystyrene‐acrylic, offering high IR emissivity and strong solar reflectivity, enabling a temperature reduction exceeding 56 °C. The effectiveness of the USRI‐enabled heat dissipation was demonstrated by its integration with a photoplethysmography (PPG) sensing system, suffering from performance degradation due to thermal buildup (Figure 9J). The USRI layer was expected to mitigate such issues by both efficient thermal radiation and non‐radiative heat transfer (Figure 9K). Temperature and PPG signal measurements across various conditions, such as indoors, in shade, and under sunlight, illustrated that the USRI‐coated device consistently maintained lower temperatures, reducing thermal fluctuations and ensuring clear and reliable biometric signal detection compared to uncoated devices (Figure 9L).

In addition to the applications discussed, recent research efforts proposed a nanofabric radiative cooler‐integrated bioelectronic system to reliably monitor stress levels in outdoor workers under extreme heat conditions.^[^ ^89^ ^]^ Additionally, transparent radiative cooling metamaterials were engineered as cover windows for foldable and flexible electronic displays, effectively mitigating thermal buildup while maintaining high optical transparency and mechanical durability.^[^ ^90^ ^]^

## 5 Conclusion and Perspectives

This review explored the diverse thermal management strategies observed in biological systems and their transformative applications in bio‐inspired technologies. Nature has evolved a vast array of sophisticated mechanisms for radiative cooling, thermal regulation, thermal insulation, moisture harvesting, IR camouflage, and IR sensing, allowing organisms to survive and thrive in extreme environments. These biological adaptations have inspired the development of innovative materials and devices, leading to breakthroughs in bio‐inspired coatings, textiles, energy‐efficient materials, and adaptive thermal management systems. **Table** **2** provides a comprehensive comparison of the structural characteristics and functional properties exhibited by diverse bio‐inspired systems. Organisms inhabiting extreme environments, such as the Saharan silver ants, camels, polar bears, and emperor penguins have provided blueprints for thermoregulatory microstructures, while Namib beetles and desert cacti have influenced advanced water‐harvesting technologies. Additionally, cephalopods and chameleons have inspired dynamic optical and thermal modulation systems for IR camouflage, while pit vipers and fire‐loving beetles have guided the design of bio‐inspired IR sensing technologies. Beyond biomimetic innovations, IR management principles have been successfully integrated into energy and electronic systems, enhancing device efficiency and stability.

**Table 2:** Comprehensive summary table of biological and bio‐inspired thermal management.

Functions	Organisms	Materials	Structures	Fabrication methods	Mechanical properties	Thermal conductivity	Solar reflectance (*R*_sol_)/solar transmittance (*T*_sol_)	IR emissivity (*ε*_IR_)/IR reflectance (*R*_IR_)	Cooling/Heating/Insulating temperature [°C]/power [W m^−2^]	Application	Ref
Radiative cooling	Saharan silver ant	PDMS, SiO_2_, Ag	Triangular prismatic structure	Nano‐imprinting	–	–	*R*_sol_ = 97.5%	*ε*_IR_ = 98%	Cooling: 6.2 °C compared to ambient air	Building, solar cell, wearable device	[8]
		PDMS	Corrugated triangular structure	Soft imprint lithography	–	0.27 W m^−1^∙K^−1^	*T*_sol_ = 97%	*ε*_IR_ = 98%	Cooling: 18.8 °C compared to bare silicon	Solar cell	[44]
		PDMS	Corrugated triangular structure	Soft imprint lithography	–	0.27 W m^−1^∙K^−1^	Δ*R*_sol_ = 17% compared to bare glass	*ε*_IR_ = 96%	Cooling: 5.6 °C compared to bare glass bottle	Wearable device	[45]
		ZnO, PDMS	Fabric with randomly embedded MCB	Coating	Tensile stress = ≈140 MPa; Young's modulus = ≈670 MPa	0.095 W m^−1^∙K^−1^	*R*_sol_ = 95%	*ε*_IR_ = 89%	Cooling:11.7 °C compared to polyester fabric	Textile	[46]
	Golden cicada	TPU, Al_2_O_3_	Microstructured film	Micro imprint	–	–	*R*_sol_ = 96.7%	*ε*_IR_ = 92.8%	Cooling: 6.6 °C compared to ambient air	Building	[28]
	Golden longicorn beetle	PDMS, Al_2_O_3_	Micropyramid‐arrayed structure with randomly embedded nanoparticles	Microstamping	–	–	*R*_sol_ = 95%	*ε*_IR_ = 96%	Cooling: 5.1 °C compared to ambient air	Electronic device, motor vehicle	[47]
	Namib black beetles	Al_2_O_3_&TiO_2_ (top) MgHPO_4_·0.78H_2_O& P(VDF‐HFP) (bottom) coated on Al sheet	Film	Spray‐coating, drop‐casting	–	–	Al_2_O_3_&TiO_2_: *R_sol_* = ≈85%	Al_2_O_3_&TiO_2_: *ε*_IR_ = ≈80%	Cooling: ≈2.9 °C compared to ambient air	Water harvesting	[65]
	Desert cacti	ZrC/PDMS on Al plate	Film	Solution casting	–	–	–	*ε*_IR_ = ≈80%	Cooling: ≈3 °C compared to ambient air	Liquid collection	[69]
Thermal regulation	Siamese cat	PVDF‐HFP, PCM	Fiber membrane	Electrospinning	–	–	Cooling mode: *R*_sol_ > 90%; Heating mode: *R*_sol_ = ≈60%	*ε*_IR_ = 95%	Cooling: 3 °C Heating: 2 °C compared to ambient air	Building, wearable device, motor vehicle	[29]
	Himalayan rabbit, mimosa	Thermochromic powder, nitrile‐butadiene rubber, Al, TiO_2_, PTHF, PCL, HDI, DBTDL	Visible‐IR thermochromic device	Blade‐coating, esterification	–	–	Cooling mode: *R*_sol_ = 65%; Heating mode: *R*_sol_ = 27%	Cooling mode: *ε*_IR_ = 95%; Heating mode: *ε*_IR_ = 28%	Cooling: 59.7 W m^−2^ Heating: 252.2 W m^−2^ compared to Al foil	Building	[53]
	Camel	TPU	Fiber with channel‐type pore	Micro‐extrusion foaming	Elongation at break = ≈275%; Tensile strength = ≈6.3 MPa	0.48 W m^−1^∙K^−1^	*R*_sol_ = 98.7%	*ε*_IR_ = 97.2%	Cooling: ≈6 °C Insulating: ≈4 °C compared to ambient air	Textile	[57]
Thermal insulation	Polar bear	Silk fibroin	Fiber with aligned pore	Freeze‐spinning	Elongation at break = ≈0.08%; Tensile strength = ≈0.95 MPa	0.022 W m^−1^∙K^−1^	–	*R*_IR_ = 70‐80%	Insulating: ≈14 °C at −20 °C	Textile	[30]
		TPU, Chitosan	Porous aerogel fiber with coating	Freeze‐spinning	Tensile strength = 12.7 MPa	0.027 W m^−1^∙K^−1^	–	High *R*_IR_ (qualitative)	Insulating: ≈10.8 °C at −20 °C	Textile	[32]
	Emperor penguin	CNT‐cellulose, PLA, MnO_2_‐cellulose	Hierarchical structure	Electrospinning, Beeswax coating	Tensile strength = 3.22 MPa	–	*R*_sol_ < 20%	*R*_IR_ = 30.8%	Heating: 7 °C compared to ambient air	Textile	[61]
Thermal management	Pelagic octopus, glass squid	Al on acrylic dielectric elastomer	Film	E‐beam evaporation	Areal strain= ≈230%	–	–	*ε*_IR_ = ≈71–≈96% depending on strain	–	Camouflage, artificial muscle, adaptive optics	[77]
	Chameleons	Ecoflex/EGaIn	Composite	Solution casting	Elastic modulus = <≈0.2 MPa, Areal strain = <≈1500%	–	–	*ε*_IR_ = 29.8 – 74.6% depending on strain		Camouflage, encoding/ decoding, sensing	[78]
	Vampire bat, pit vipers	UCNPs & HEK293‐ChR2 on graphene	–	Solution casting	–	–	–	–	–	IR sensor	[82]
	*Melanophila acuminata*	PI/carbon bilayer, PDMS/PDA composite, PMMA chamber	–	Laser cutting, solution casting, transfer‐printing	–	–	–	–	–	IR sensor	[85]

Despite significant progress, several challenges and opportunities remain in translating bio‐inspired thermal management technologies into practical applications. A critical challenge in the commercialization of bio‐inspired thermal management technologies lies in achieving sustainable and scalable fabrication of complex micro‐ and nanostructures. Manufacturing techniques such as lithography and electrospinning, while effective in replicating biological architectures with high precision, are often constrained by limited throughput, high production costs, and challenges in maintaining structural uniformity over large areas. These limitations cause significant barriers to large‐scale production and real‐world applications. To overcome these challenges, the development of cost‐effective and high‐throughput fabrication strategies is essential. Emerging technologies such as 3D printing,^[^ ^91^, ^92^ ^]^ self‐assembly,^[^ ^93^, ^94^ ^]^ and roll‐to‐roll processing^[^ ^95^, ^96^ ^]^ offer promising pathways toward scalable manufacturing. For example, 3D printing allows for customizable and precise fabrication of complex geometries, enabling the replication of intricate biological structures. Self‐assembly techniques leverage spontaneous molecular organization to form ordered structures over large surface areas at low cost. Roll‐to‐roll processing facilitates continuous manufacturing, making it particularly suitable for producing flexible films and large‐area coatings. Incorporating these scalable approaches into bio‐inspired thermal management systems can bridge the gap between laboratory‐scale demonstrations and industrial applications. Additionally, current bio‐inspired designs often struggle to match the precision and efficiency of their natural counterparts, particularly in IR camouflage and sensing. Advancing fabrication technologies that incorporate biohybrid materials will be key to enhancing performance, adaptability, and precision.

Another critical factor in the practical deployment of bio‐inspired thermal management systems is ensuring durability and environmental stability, particularly for materials intended for outdoor or extreme environmental conditions. Addressing these issues requires the development of materials capable of withstanding prolonged exposure to heat, humidity, mechanical stress, and UV radiation while maintaining their functional integrity. This involves exploring advanced surface treatments and protective encapsulation techniques to enhance environmental resilience. Moreover, successful integration with energy and electronic systems poses engineering challenges, particularly thermal expansion mismatch. For instance, recent studies have demonstrated that applying radiative cooling layers to PV modules can significantly reduce surface temperatures, thereby enhancing power output while extending device lifespan.^[^ ^86^ ^]^ However, thermal expansion mismatches between cooling materials and PV cells can induce mechanical stress, leading to potential delamination or cracking under cyclic thermal loads. Addressing these challenges will require innovative interfacial design strategies, such as graded thermal expansion layers or interfacial adhesives to accommodate differential thermal expansion without compromising device stability.

Sustainability is also a growing concern, highlighting the need for biodegradable and recyclable materials in IR management systems to further support energy conservation and climate adaptation. In this respect, future research should prioritize sustainable polymers like PLCL,^[^ ^97^ ^]^ poly(glycolide‐co‐ε‐caprolactone) (PGCL),^[^ ^98^ ^]^ poly(glycerol sebacate) (PGS),^[^ ^99^ ^]^ and poly(butylene succinate) (PBS),^[^ ^100^ ^]^ which offer biodegradability, high IR emissivity, and mechanical resilience. Integrating natural fillers, such as cellulose nanofibers and chitosan, can further enhance mechanical strength and thermal stability while supporting circular economy principles.^[^ ^101^ ^]^ In addition, AI‐driven material design presents transformative opportunities to accelerate the discovery and optimization of sustainable IR management materials. Machine learning models can rapidly predict material properties, optimize microstructural designs, and simulate thermal performance, significantly reducing development time. Generative design algorithms further enable the creation of novel bio‐inspired architectures optimized for radiative cooling and thermal regulation.

Finally, many biological species remain unexplored in terms of their IR‐related adaptations. A systematic investigation of understudied biological systems, including plants, deep‐sea organisms, and nocturnal species, could unveil novel strategies for IR manipulation, expanding the possibilities for next‐generation biomimetic materials. By addressing these challenges, bio‐inspired thermal management technologies can evolve into scalable, durable, high‐performance, and sustainable solutions for energy‐efficient applications and innovative advancements across diverse fields.

## Conflict of Interest

The authors declare no conflict of interest.
